# Well-being Messaging for Mammalian Milks: A Scoping Review

**DOI:** 10.3389/fnut.2021.688739

**Published:** 2021-10-22

**Authors:** Saleh Moradi, Joanne Hort

**Affiliations:** ^1^Food Experience and Sensory Testing (Feast) Laboratory, Massey University, Palmerston North, New Zealand; ^2^Riddet Institute, Massey University, Palmerston North, New Zealand

**Keywords:** well-being, food label, health claim, health communication, milk, scoping review, well-being messaging

## Abstract

Having a holistic understanding of research on well-being messaging for milk is vital to allow the optimal communication of the association between milk consumption and various nutritional, physical, and psychological benefits to the consumer. This work is a unique interdisciplinary, scoping review of existing research on well-being messaging for milk. Well-being messages are ways to communicate the broad well-being benefits of specific products to the consumer through information on food content or statements that link a product with favourable components, functions, or well-being outcomes. Leveraging this broad definition, and by proposing a guiding theoretical model that considers well-being messaging as a form of communication, milk well-being messaging literature has been mapped across time, geographical locations, disciplines, and product types. Two hundred forty-six were records included in this review, of which 177 were empirical studies. Studies were disseminated between 1954 and 2019, with 54.9% published after 2011. Food, Agriculture, and Biological Sciences (*N* = 109), Nutrition and Dietetics (*N* = 78), and Medicine, Public Health, and Health Professions (*N* = 69) disciplines have attracted the most publications, with numbers generally increasing in most recent years. The majority of included non-empirical records (69.6%) provide lists of commercially available products carrying well-being messaging and/or regulations on the use of particular well-being messages for milk according to various legislative authorities. Most of the empirical studies were conducted in North America (*N* = 71), West Europe (*N* = 52), and Oceania (*N* = 22), and on plain (i.e., unflavoured) milk (*N* = 152). Whereas, most studied elements of well-being messaging for milk across time, i.e., *message* (*N* = 169), *product* (*N* = 141), *receiver* (*N* = 101), and *context* (*N* = 72) have seen an increasing number of studies in recent years; *sender* (*N* = 51) and *medium* (*N* = 27) have been even less studied in the past four years. A more detailed analysis of research trends in each element of well-being messaging is reported. The research highlights immediate and strategic knowledge gaps that need further attention from researchers and/or policymakers in order to improve the “messaging” of well-being benefits of milk consumption to the consumer.

## Introduction

Whether looking at ancient mythologies or the documented history of mankind, there are few food products that appear consistently, milk is undoubtedly one of them. Milk, exclusively defined as the normal mammary secretion obtained from milking of mammals, including but not limited to bovine (1), for millennia, has been regarded as an integral part of human diet in most parts of the world. It has most commonly been regarded as a nutritious food with various health benefits. Despite some polarising debates emerging in recent years, mounting evidence from various research disciplines continues to support the unique nutritional value of milk (1–3) and an association between milk consumption and many physical and also psychological benefits. A non-exhaustive list of the well-being domains that milk consumption has been positively associated with reduced incidence of colorectal, bladder, and gastric cancer reduced risk of obesity, diabetes, cardiovascular disease, frailty, and sarcopenia, and improved metabolism, bone health, gut function, and cognitive performance (1, 4–6).

The association between milk consumption and well-being, no matter how scientifically established, can only reach the consumer if it is communicated accurately and effectively to the public. Informing consumers of the health and nutritional value of milk has, indeed, been shown to influence milk consumption behaviour, purchasing behaviour, and willingness to pay (7–11). Food labels have, for a long time, been considered to inform the consumer about well-being effects of food products (12, 13). Surveys in Western countries suggest that food labels are relatively popular sources of information among the general population and more so among certain market segments, including women, higher-income earners, younger consumers, Caucasians, weight-conscious, or worried-well (see 12, for a review). Delivered at the point of choice, purchase, or consumption, food labels can be of various types (e.g., mandatory vs. voluntary), frames (e.g., promoting vs. warning), designs (e.g., pictorial vs. textual), content (e.g., nutrition, health, or risk reduction), and strengths (e.g., qualified health claims vs. marketing information). Fat content of milk, to give an example, has traditionally appeared on a package (e.g., “low fat,” “skim milk,” and “full cream”). Recent advancements in dairy science and technology (e.g., developing functional foods with specific health effects), as well as marketing and retailing approaches (e.g., growing popularity of online supermarkets or social media marketplaces), have certainly added more sophisticated front-of-pack (FOP) profiling systems and QR codes to traditional food labels. These new forms of information, while ultimately performing as means to communicate well-being messages of a product to the consumer, have commonly been targeted as separate lines of research across various disciplines and, sometimes, with terminology and scope that do not include other forms of messaging. Hence, despite existing reviews of literature on either nutrition labels (14), health-related claims (15), point-of-choice messaging (7), and FOP labels (16) for food products, no effort has, to date, been made to map relevant literature on milk well-being messaging regardless of the type, frame, design, content, strength, or medium of a message.

This review aims to bring together a seemingly diverging literature around what is essentially *well-being messaging* for milk regardless of species. This is achieved through the provision of an all-encompassing definition of well-being messaging and proposition of a guiding theoretical model that considers well-being messaging as a form of communication. Together, these make it possible to map the literature on well-being messaging for milk across time, geographical locations, disciplines, and product types. The research, therefore, highlights immediate and strategic knowledge gaps that need further attention from researchers and/or policymakers in order to improve “messaging” the well-being benefits of milk consumption to the consumer.

### Well-being Messaging

While a commonly used term in the literature is “health,” well-being brings about a broader connotation that covers not only the state of being physically healthy but also potential psychological and social benefits that are offered by consuming food. Fittingly, well-being, as a multifaceted concept, is defined as a way of living that directs one toward the highest levels of feeling good and functioning well in various biopsychosocial domains (17–19). Interestingly, prior research highlights the association between milk consumption and various psychological and social domains (20–23), hence justifying the need for the use of the term “well-being” instead of “health” in relation to milk.

With a definition of well-being stated, well-being messaging can largely be considered as communicating well-being-related information. To provide a working definition, however, well-being messaging is defined as ways to communicate to the consumer the broad well-being benefits of specific products through information on food content or statements linking the product with favourable components, functions or well-being outcomes [for a comparison, see (24)]. Being broad enough, this definition fits the purpose of this research to include instances of well-being messaging for milk regardless of type, frame, design, content, strength, or medium.

### Theoretical Framework

Review studies benefit from a predetermined conceptual framework, which is anticipated to best encompass the broad questions of interest (25, 26). Having a conceptual framework is particularly useful as a basis for scoping the boundaries of the review (e.g., inclusions and exclusions), initial coding, and categorising the existing evidence. A framework also helps to identify patterns, trends, and themes in prior research and thereby highlights knowledge gaps in the literature (25–27). This so-called “best fit” framework has also been suggested to offer promising responses to practise-relevant and policy questions about the topic of interest (27).

For the purpose of this review, Lasswell's communication model, a classic model of communication, has been chosen as a framework for mapping out milk well-being messaging literature. According to this model (28), any act of communication is comprised of five essential elements: sender (i.e., who said it), message (i.e., what was said), medium (i.e., through what channel it was said), receiver (i.e., to whom it was said), and outcome (i.e., what was the desired outcome). Fittingly, a well-being message is a *message* that is sent by a *sender* (e.g., food manufacturer) through a specific *medium* (e.g., on or off the food package) to facilitate an anticipated *outcome* (e.g., increased well-being awareness or willingness to purchase) in *receiver(s)* (e.g., a particular market segment).

To the extent that Lasswell's original model is criticised for ignoring the context in which the communication takes place, a limitation that has been addressed in subsequent theories of communication [e.g., (29)], a well-being message, as the specific communication type of interest here, is also highly contextualised. As such, legislative zones, geographical markets, and/or selling venues are likely to influence (or even enforce) the decision of a manufacturer with regard to various aspects of the well-being messaging. Also, despite the fact that “medium,” in accordance to Lasswell's model, can broadly include the product itself as a channel that delivers the message, to provide further practicality and clarity around categorisation of published research and identification of knowledge gaps, product, and medium have been considered as separate elements of communication in this review.

Adding context and product to the original model, it is proposed that well-being messaging studies conducted in any discipline are ultimately engaged in investigating one or more of these major elements: product, message, sender, receiver, medium, context, and outcome. The remainder of this section provides a working definition of each element together with instances of each that have been incorporated in categorising records in this scoping review.

*Product* is milk (i.e., including milks of all mammalian origins, but excluding plant-based imitation milks) and other milk drinks included within the scope of this review. Product-related research thus includes any instances of product characteristics (e.g., price, total fat, sugar, calories, and other nutrient contents, size, packaging design, or material) when studied in association with well-being messaging.

*Message* simply refers to the information that is conveyed through the well-being message. Hence, studies that have investigated the content and/or the graphical and verbal design of well-being messaging have been categorised under this element.

*Sender* denotes the source of the well-being message. The sender is usually the food manufacturer. As such, any piece of evidence examining manufacturer-related factors (e.g., brand, image, size, and country of origin), as well as organisation-related decisions and procedures involved in (or affected by) development, design, use, and/or (re-)evaluation of well-being messaging, has been categorised under this element. In addition, endorsements and verifications of the well-being message by sources other than the manufacturer have also been considered as instances of sender related.

*Receiver* of the well-being message is generally the consumer. Well-being messages, however, are sometimes used to target a particular segment of the market, determined by factors such as consumer age, particular health concerns, socioeconomic status, or social group. Studies that have investigated consumer-related factors (e.g., demographics, diet and eating habits, lifestyle, health status, and bodily measures), whether individually or in order to create clusters of consumers, in relation to well-being messaging, have thus been categorised as receiver related. It is important to note that records wherein participant information is only mentioned in descriptive statistics and not in association with well-being messaging have not been considered as instances of receiver-related research.

*Medium* refers to the exact channel of well-being message delivery. While the usual medium of delivering well-being messaging is the food package, well-being messages are sometimes delivered off-the-pack (e.g., TV commercials, online shopping platforms, or supermarket displays). Instances of empirical research analysing placement of well-being message, delivery format and design, point of choice delivery, marketing strategies to boost well-being messaging, and advertisements have all been considered as medium-related research.

*Contexts* in which well-being messages are used may be analysed at various levels, e.g., selling point, time and date, geographical market, and legislative zone. Factors relevant to each level, when analysed in relation to well-being messaging, have been counted as instances of context-related research. It should, however, be noted that investigations of self-identified social groups (e.g., religious or ethnic group) are considered instances of receiver-related research.

Well-being messaging aims at, and possibly delivers, various *outcomes*. Food manufacturers, for instance, use well-being messages with the ultimate goal of increasing their market sales, perhaps through enticing segments in the market who would most likely benefit from consuming the product. This outcome, when considered from a consumer point of view, is usually translated as purchasing and consumption behaviour. Various outcomes of interest in milk well-being messaging research, whether related to design, delivery, or perception of well-being message or as a result of utilising well-being messaging, have been included here.

It is also worth noting the inclusive approach that has been taken in categorising evidence under the elements listed above.

### Research Questions

Within the presented theoretical framework and stated definition of well-being, this review aims to answer the following research questions (RQ):

RQ1: What are the identifiable research trends (across scientific disciplines, time, geographical locations, product type, and research methods) regarding well-being messaging for milk?

RQ2: Akin to the proposed conceptual framework, what well-being messaging elements have received more attention across scientific disciplines, time, and geographical locations than others?

RQ3: What have been the specific areas of interest within each well-being messaging element across time and for various product types?

RQ4: What have been the specific outcomes of interest within each scientific discipline?

## Methods

Nature, scope, and existing knowledge around a scientific enquiry are the keys when deciding the correct approach to address it. Given the form of our research questions, multidisciplinary nature of the area, the expected heterogeneity of research methods, and inability to find comprehensive reviews of the topic through a preliminary search, a scoping review was chosen as the appropriate method. A scoping review is “a type of knowledge synthesis, following a systematic approach to map evidence on a topic and identify main concepts, theories, sources, and knowledge gaps” (30, p. 467). Scoping reviews are mainly aimed at painting a big picture of the extent, range, and pattern of research activity in a topic area of a particular complex, interdisciplinary, or heterogenous nature, clarify key concepts and identify knowledge gaps in the existing literature, and determine the value and potential scope of undertaking a systematic review (30, 31). The methodology and the reporting agenda in this study are adapted from suggestions by Pham et al. (31), Arksey and O'Malley (32), Levac et al. (33), and Tricco et al. (34) in an effort to follow the best practise guidelines in the literature while accommodating the specificities of our research.

### Identification of Relevant Studies

Relevant literature for this review was extracted from a database of research studies on well-being messaging for food products systematically developed by the authors. Relevant studies cited, but not originally included in the database, were also added to the pool of identified literature for final screening. An account of how this comprehensive database was developed and relevant studies identified is provided below.

#### Database of Well-being Messaging Studies for Food Products

The database systematically mapped interdisciplinary literature around well-being messaging for food products, including but not limited to milk. It was developed following guidelines in the literature (35, 36) and according to a detailed preregistered protocol, which is accessible *via* the open-science framework repository (37).

##### Data Sources and Search Strategy

EBSCo (Academic Search Complete, Art and Architecture Complete, Business Source Complete, Cinahl, EconLit, Education Source Complete, MEDLINE, PsycInfo, and PsycArticles), Scopus, Web of Science, Cochrane, and ProQuest were searched between 23–31 December 2019 for academic journals, early access, reviews, conference materials, editorial material, books, e-books, book chapters, and dissertations published in English up to and including December 2019. Given the interdisciplinary nature of the enquiry, the data sources were selected to be comprehensive and to cover a broad range of disciplines. The search query of titles, abstracts, and keywords, while being tailored to the specific requirements of each source, consisted of the following sets of terms: all combinations of type terms (e.g., “health,” “wellness,” and “well-being”) and communication terms (e.g., “claim,” “label,” and “message”), topic terms (e.g., “food,” “product,” and “consume”), but excluding irrelevant terms (e.g., “insurance,” “health information technology,” and “health monitor”). The search query was informed by *a priori* knowledge, examining recent relevant systematic reviews, and following pilot searches. The search query for Scopus is provided as an example in the preregistered protocol (37).

##### Eligibility Criteria

Studies were included if they were: (i) document types limited to academic journal articles, doctoral dissertations, book and book sections, indexed reports, and conference papers; (ii) involving any research designs and methodologies, including empirical and non-empirical records (e.g., reviews, theoretical pieces, letters, and opinion papers); (iii) records with full texts obtainable through institutional holdings available to the authors or published in open-access outlets; (iv) records with full text in English (It should be noted that, despite excluding the records with non-English full texts, no limitations have been set regarding the country in which the study was carried out or where it has been published); (v) records attending to well-being messages of all types and formats, with the notable exceptions of an ingredients list and a nutrition information table; (vi) involving well-being messaging for pre-packaged food products, excluding alcoholic drinks, tobacco products, unpackaged food, menus, drugs, dietary, nutritional, herbal, and medical supplements, nutraceuticals, and the like.

##### Citation Management

Citations were imported into Mendeley Desktop 1.19.4, and duplicate citations were removed manually. Citations were then imported into Rayyan data management software for screening (38). Finally, selected citations were imported into Endnote X9 with full texts added automatically *via* the “find full text” feature, or manually. Further duplicates were removed when found during the screening process, manually, or using Endnote X9.

##### Screening Process

Records were screened for eligibility in Rayyan, first based on title only, and then abstract and keywords. In rare cases, full texts were consulted during the prescreening step. Following prescreening, a further screening examined document-type eligibility, full-text language, and full-text accessibility on Endnote X9.

The screening process, conducted by the first author, followed a predetermined procedure (outlined in detail in the preregistered protocol) to mitigate assessor bias. Specifically, after transferring search results to Rayyan, a random subsample of 100 results was created by the first author and transferred to a separate pilot screening project in Rayyan. This subsample was blindly screened based on title, abstract, and keywords by both authors. The first screening round resulted in 77% inter-rater agreement and did not reach the predetermined 80% threshold. Hence, after discussing cases of disagreement, a second screening was conducted and resulted in 93% agreement.

The procedure outlined above resulted in 3,337 records being included in the general well-being messaging database (see Supplementary Figure 1).

#### Identifying Relevant Studies for the Scoping Review

To identify studies focusing on well-being messaging for milk, the database was searched for the following three steps: (i) full texts of all included records were prescreened to separate searchable and unsearchable PDF files; (ii) searchable PDF files (*N* = 3,255) were automatically searched using the search function of Adobe Acrobat Reader for variations of the term “milk” (e.g., “milks,” “milkshake,” and “milkfat”). Records including any variation of “milk” were considered for further screening; (iii) all unsearchable PDF files (*N* = 82) were also considered for further screening.

Additional studies relating to well-being messaging for milk, whether cited in-text or in reference lists of the database records, were sourced and added to the pool of records for further screening (*N* = 83).

In total, 3,420 records were identified for screening for the scoping review.

### Scoping Review Study Selection Process

#### Additional Eligibility Criteria

As well as the well-being messaging database eligibility criteria (listed in section Eligibility Criteria), the following additional criteria were added: (i) records pertaining to well-being messages for all types of pre-packaged ruminant milks, including pourable, powdered, and flavoured milk, functional non-fermented milk, and milkshake, but excluding infants and toddlers milk formula, nondairy milk substitutes, and fermented milk drinks were included; (ii) records in which findings exclusive to milk or other product types of interest were not separated from other products (e.g., dairy products, milk products, milk and other dairy products, milk and other soft drinks, and milk and other milk alternatives) were excluded in the screening process.

All identified records were imported into Endnote X9 and full texts were screened against the eligibility criteria. To reduce assessor bias, a random subsample of records in the well-being messaging database (98 records out of 3,255 searchable PDF files) was blindly searched for and screened for eligibility by both authors. Inter-rater agreement of 91% was achieved (> the predetermined 80% threshold), cases of disagreement were discussed, and the first author continued with screening. The screening consisted of two steps. The first step (i.e., prescreening) excluded 3,073 searchable PDF records based on the fact that an automated text search for any variation of “milk” did not return eligible results. This step identified any mentions of milk and its variations anywhere in the record and made it possible to evaluate the eligibility of the record without the need to go through the full text. Cases for which a clear decision could not be made *via* this process were considered for further screening (i.e., the second step).

**Figure 1:**
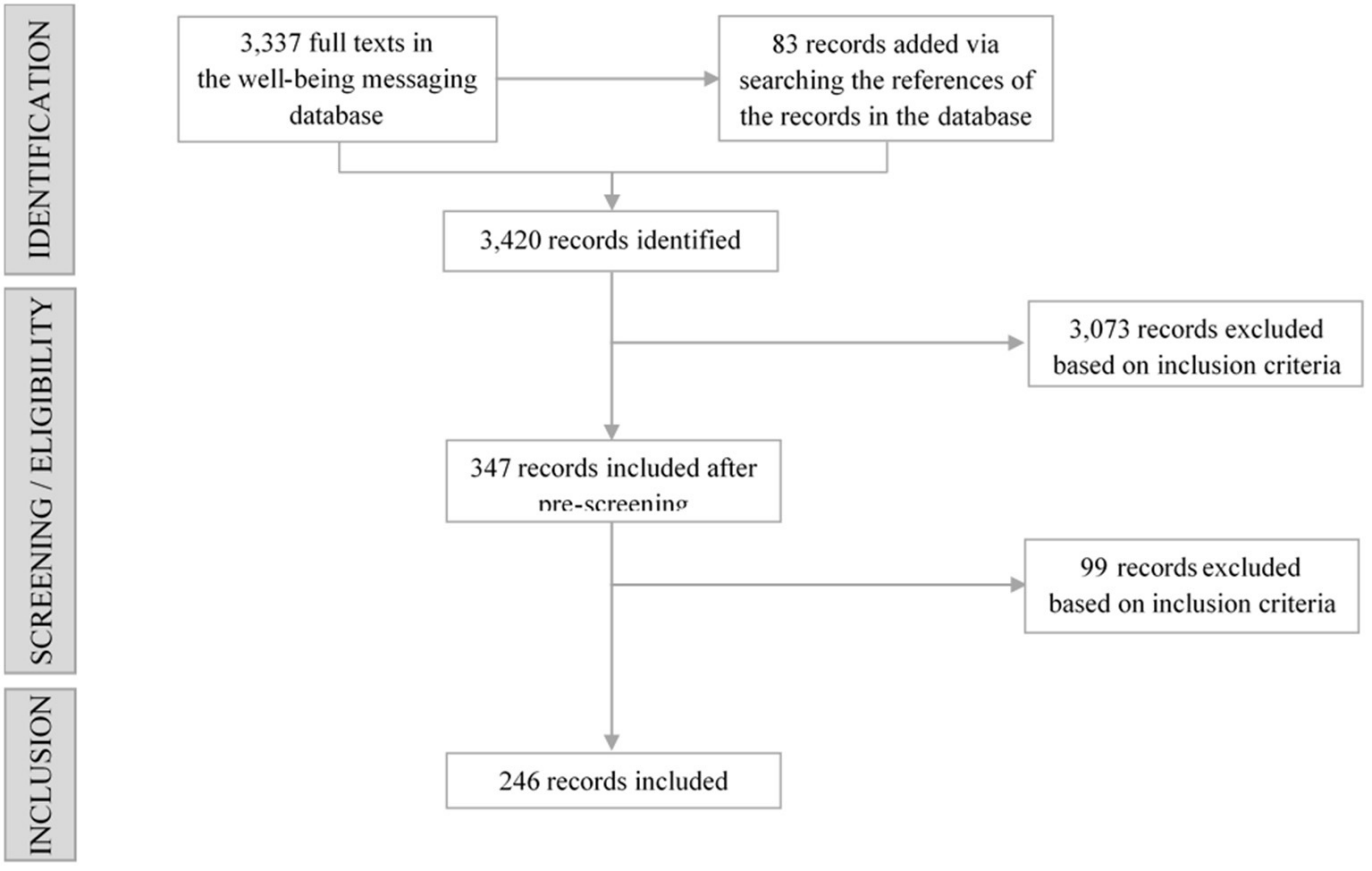
A search flowchart resulting in the final 246 records included in this review.

The second step involved screening the remaining 347 records that either the file format did not allow for automated text search or a final decision for eligibility was deemed difficult after the prescreening step. Either case, the full text was manually screened for relevance and eligibility against the inclusion and exclusion criteria. This resulted in 246 records (the list obtainable on request *via* feast@massey.ac.nz) for inclusion in this scoping review (see Figure 1).

### Charting the Data

Data charting was completed with an *a priori* coding framework (see Supplementary Table 1) focused on time of publication, publication outlet, and its associated scientific discipline(s), record type for both empirical and non-empirical records, and research method, study design, product type, country(-ies) of study, well-being messaging elements of focus (i.e., product, message, sender, receiver, medium, and context), outcome(s) of interest only for empirical records. Non-empirical records, however, were also accompanied by a brief summary of content and were classified as one of the following publication types: reviews (which include meta-reviews), case reports, conceptual pieces, or opinion papers. Given the multidisciplinary nature of the review and variety of designs employed, “study design” was dropped from the framework as accurate identification and reporting were deemed to be difficult. Publication years and geographical locations were categorised into time periods of 1954–2000, 2001–2005, 2006–2010, 2011–2015, and 2016–2019; and geographical regions as North America, Latin America, West Europe, East Europe, West Asia, East Asia, Africa, and Oceania following Maddison (39). Scientific discipline(s) of each record were determined *via* journal-associated disciplines identified *via* Scimago Journal and Country Rank (https://www.scimagojr.com) and further grouped into 19 disciplines (see **Figure 3**). Records published in outlets other than academic journals were classified as “unspecified” discipline. Research methods were classified as quantitative, qualitative, and mixed methods. Product types were classified as plain (i.e., unflavoured) milk (fluid), plain milk (powder), flavoured milk, milkshake, others. Unspecified product types were classified as plain milk (unspecified). Finally, it is worth noting that, for records including empirical and non-empirical sections, multiple studies, various methods, or more-than-one-product type or a well-being messaging element of focus, parts that met the eligibility criteria were used for charting the data. Similarly, among the variables of study, only those studied in connexion with well-being messaging were used to chart the data. Hence, study participant information, for instance, was only considered to be an example of a receiver-related study when investigated in association with well-being messaging.

## Results

### General Trends

**Figure 2:**
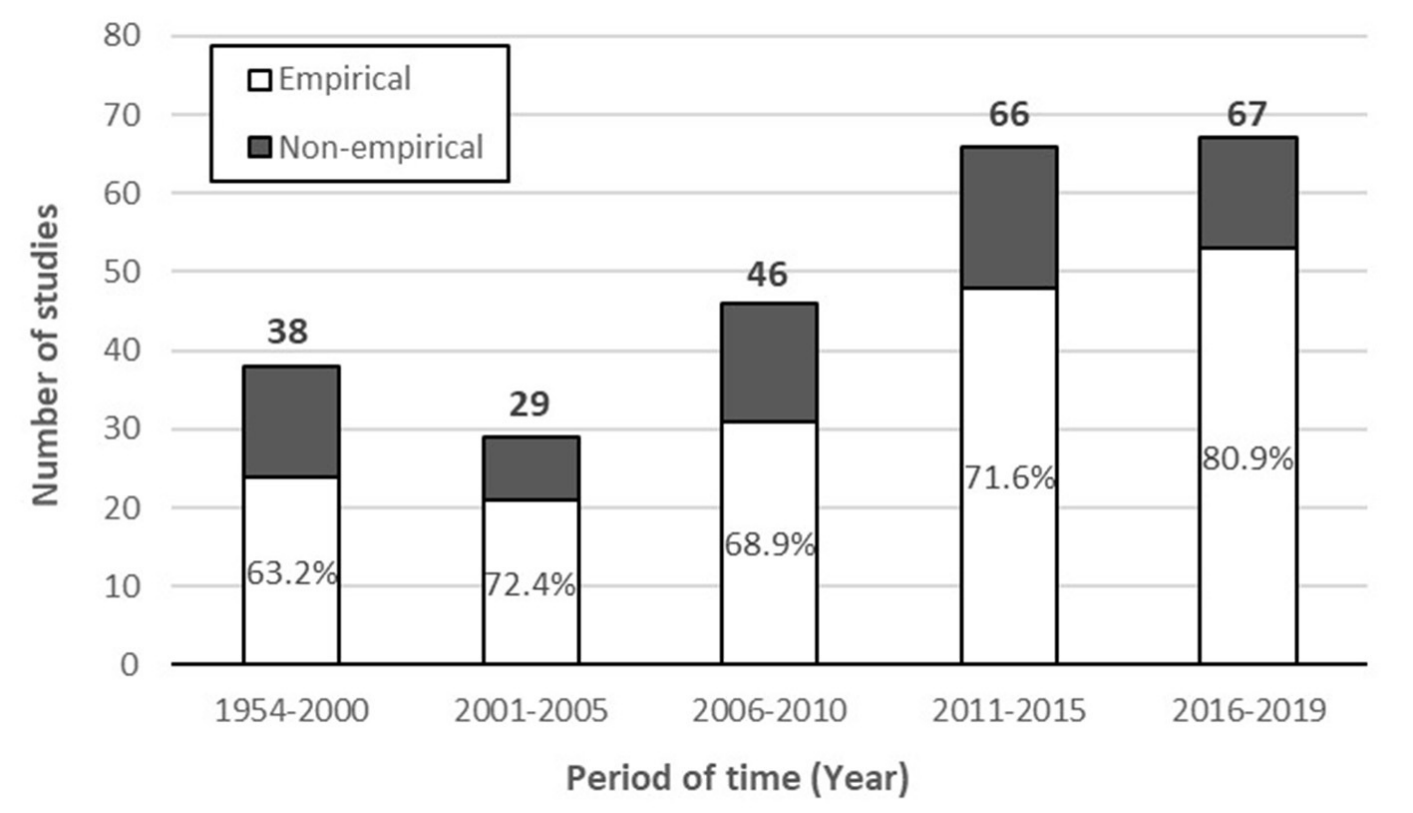
Prevalence of empirical and non-empirical studies of well-being messaging for milk during each period (i.e., 1954–2000, 2001–2005, 2006–2010, 2011–2015, and 2016–2019). Numbers over each bar represent the total count of studies during each period. Empirical studies as a percent of total have been indicated inside the bars.

Out of the 246 records included in this review, 177 records were classified as empirical (72%), and 69 as non-empirical (28%). The ratio of empirical to non-empirical records, however, has been consistently increasing in recent years (Figure 2). While only 63.2% of records published between 1954 and 2000 were empirical, the percentage increased to 80.9% between 2016 and 2019.

Empirical records were composed of 167 journal articles (94.3%) with the remainder book chapters (1.1%), conference papers (1.7%), PhD theses (0.6%), and published indexed reports (2.3%). The majority of empirical records took a quantitative approach (87%). Qualitative and mixed methodologies were used in 7.9 and 5.1% of the empirical records, respectively. With regard to product type(s) of interest, plain milk was the most commonly studied product type (152 records) compared with flavoured milk (31 records), milkshake (8 records), and other milk drinks (7 records). Among plain milk records, however, powdered milk has received much less attention (5 records) compared with fluid milk (56 records). It is worth noting that, in a considerable number of the records investigating plain milk, the format (fluid vs. powder) of the product was not specified (91 records). It should also be noted that some records had more than one type of product; hence, the counts presented are mutually inclusive. See Table 1 for a more detailed breakdown of the empirical records by record type, research method, and product type.

**Table 1:** A summary of empirical and non-empirical records by record type, method, product type, and publication type.

**Empirical (177)**			**Non-empirical (69)**	
**Record type**	**Method**	Product type^*^	**Record type**	**Publication type**
Journal article (167)	Quantitative (145)	Plain milk (fluid) (46)	Journal article (51)	Review (42)
		Plain milk (powder) (4)		Case report (3)
		Plain milk (unspecified) (71)		Conceptual piece (3)
		Flavouredmilk (29)		Opinion paper (3)
		Milkshake (8)	Book Chapter (14)	Review (14)
		Others (6)	Book (2)	Review (2)
	Qualitative (14)	Plain milk (fluid) (1)	Conference paper (2)	Review (2)
		Plain milk (powder) (1)		
		Plain milk (unspecified) (11)		
		Others (1)		
	Mixed methods (8)	Plain milk (fluid) (3)		
		Plain milk (unspecified) (5)		
		Flavouredmilk (1)		
Book chapter (2)	Quantitative (1)	Plain milk (fluid) (1)		
	Mixed methods (1)	Plain milk (unspecified) (1)		
Conference paper (3)	Quantitative (3)	Plain milk (fluid) (3)		
		Flavouredmilk (1)		
Thesis (1)	Quantitative (1)	Plain milk (fluid) (1)		
Indexedreports (4)	Quantitative (4)	Plain milk (fluid) (1)		
		Plain milk (unspecified) (3)		

*Counts are shown between brackets*.

* *Some records investigated more than one type of product*.

Non-empirical records comprised 51 journal articles (73.9%), with the rest being book chapters (20.3%), books (2.9%), and conference papers (2.9%). Review studies, including narrative, systematic, and meta-reviews, accounted for almost 87% of the entire non-empirical records included in this study, with case reports, conceptual pieces, and opinion papers, each representing up to 4.3%. Table 1 provides a detailed breakdown of non-empirical records by record type and publication type.

**Figure 3:**
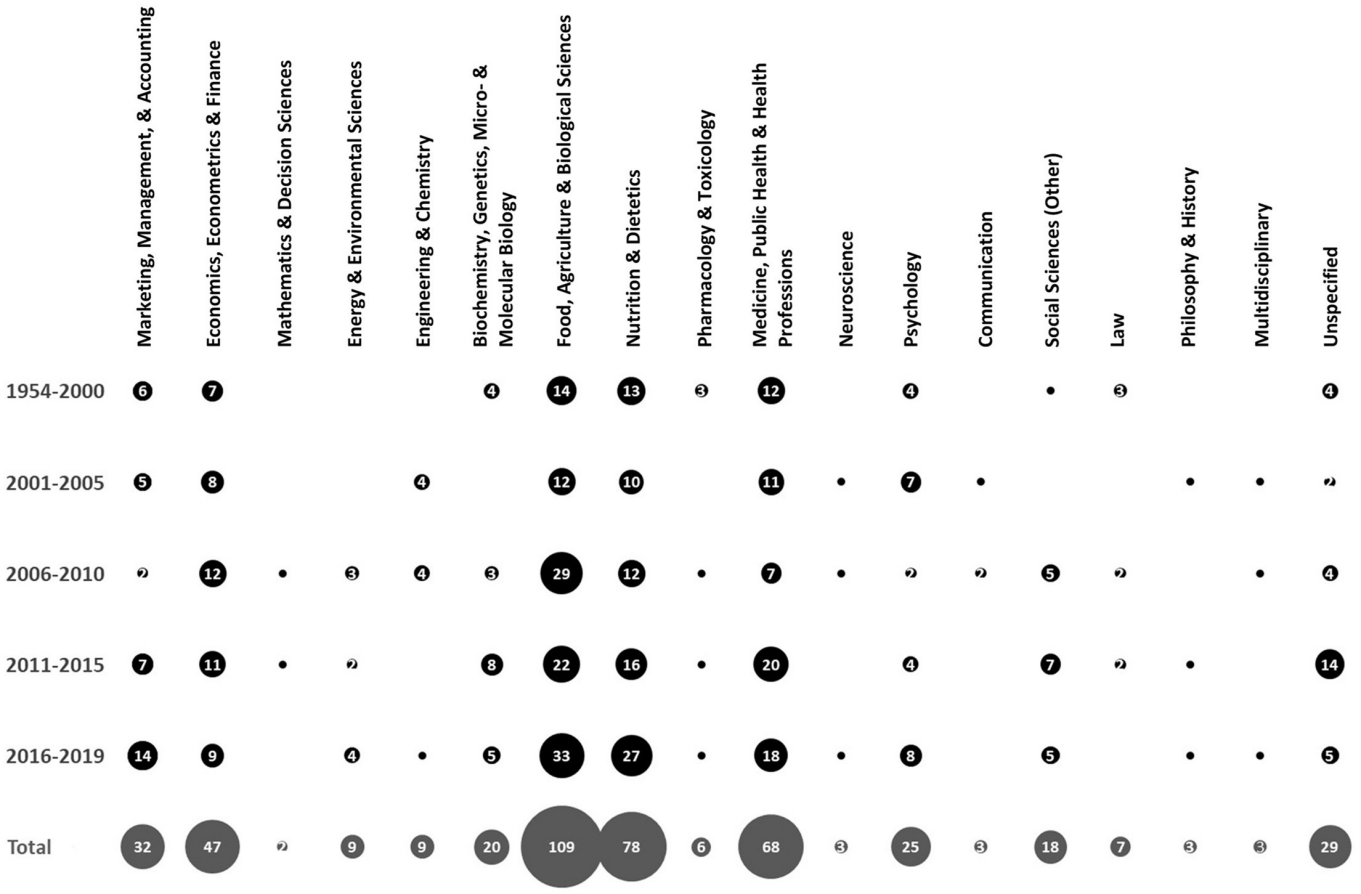
Prevalence of research in each discipline during each period (i.e., 1954–2000, 2001–2005, 2006–2010, 2011–2015, and 2016–2019) and in total (i.e., between 1954 and 2019). Numbers in bubbles specify the prevalence. Note that bubbles with no number represent only one record.

A further breakdown of records by time and academic discipline(s) of publication is presented in Figure 3. While accepting that each record may have been assigned to more than one academic discipline, the following notable trends could be observed: (i) Food, Agriculture, and Biological Sciences, Nutrition and Dietetics, Medicine, Public Health, and Health Professions, respectively, have attracted the most publications, with numbers generally increasing in most recent years; (ii) Economic, Econometrics, and Finance, Marketing, Management, and Accounting, Psychology, and Biochemistry, Genetics, Micro- and Molecular Biology have each been involved in at least 20 publications across time, albeit with somewhat different temporal trajectories; (iii) while publication numbers in several disciplines, e.g., Energy and Environmental Sciences, and Social Sciences (Other) have been growing in the past 15 years, disciplines like Engineering and Chemistry, Communication, and Law have not been involved in publishing research in well-being messaging for milk in the past few years.

### Overview of Non-empirical Literature

While this review is set to provide a detailed account of empirical records, this section features a brief yet unique overview of non-empirical records, which serves three key purposes: First, to inform the reader about the nature, scope, subject, and methodology of previously published reviews; second, unlike disciplines, such as Communication, Neuroscience, and Social Sciences, where the included records were all classified as empirical, milk well-being messaging research in Law was confined to non-empirical studies, including two original pieces (case report and opinion paper), which would be of interest for readers within the discipline or across other disciplines; third, the majority of included non-empirical records (69.6%) provided lists of commercially available products carrying well-being messaging and/or regulations on the use of particular well-being messages for milk according to various legislative authorities, which are both of key interest to industry and policy-makers.

Given the above, a summary of non-empirical records has been provided in the (Supplementary Table 2) that can be used to assess record and publication types, and the main themes of non-empirical research throughout the time span of this review. It should be noted that none of the reviews provided either a scoping review of well-being messaging for milk or an interdisciplinary approach to the topic, hence adding to the importance of this research as a unique contribution to the existing literature. Also, a closer look at the most recent nonempirical records (i.e., 2016–2019), it is clear that: (i) 10 out of 14 records published in this period were journal articles with the rest being book chapters; (ii) except for one case report published in 2018, all other non-empirical records have been categorised as reviews; (iii) the main theme of the included records content-wise related to food-related regulatory systems in different legislative zones, especially with regard to well-being messaging for milk (8 records), lists of commercial milk across the globe carrying well-being messaging (3 records), impacts of well-being messaging, among other interventions, on milk consumption and willingness to pay for milk (3 records), and a review of product- and consumer-specific characteristics influencing the impact of well-being messaging for food products, including milk.

### Overview of Empirical Literature

**Figure 4:**
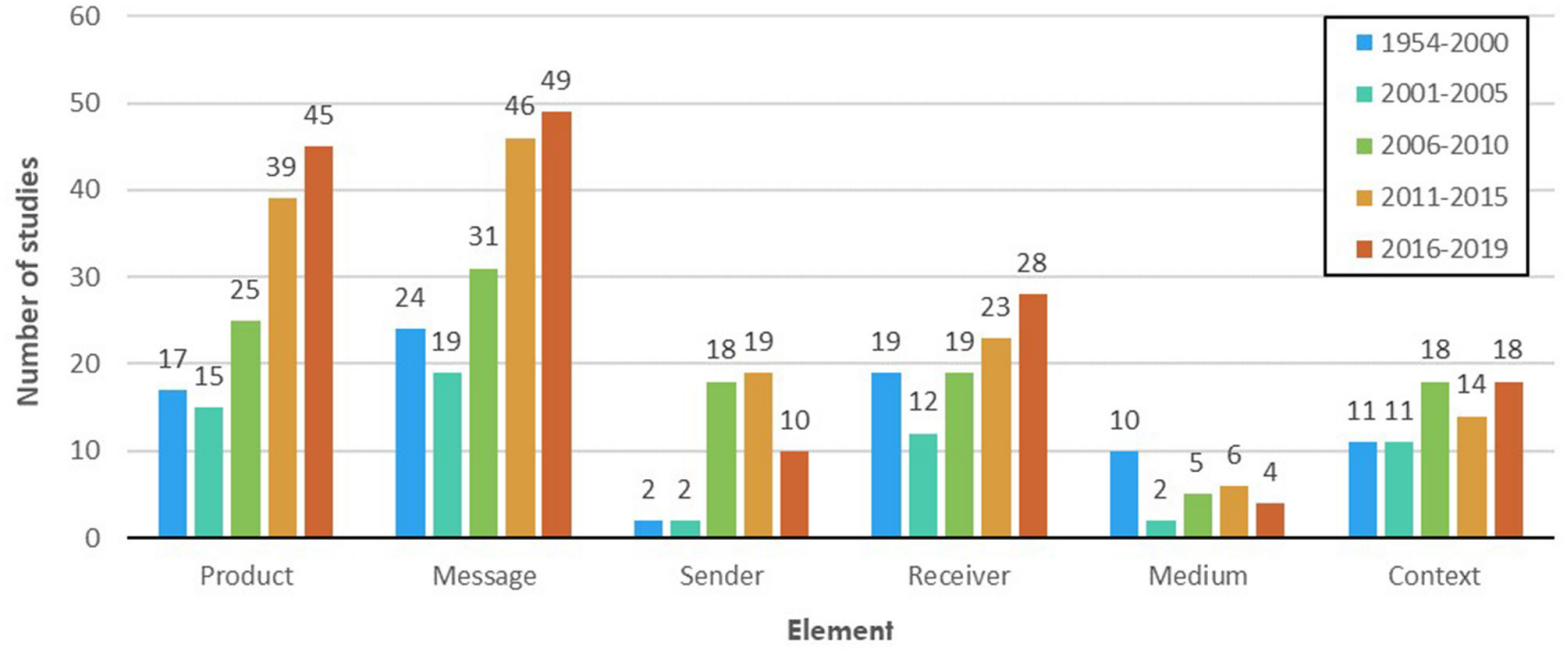
Prevalence of empirical studies of each element (i.e., product, message, sender, receiver, medium, and context) during each period (i.e., 1954–2000, 2001–2005, 2006–2010, 2011–2015, and 2016–2019).

**Figure 5:**
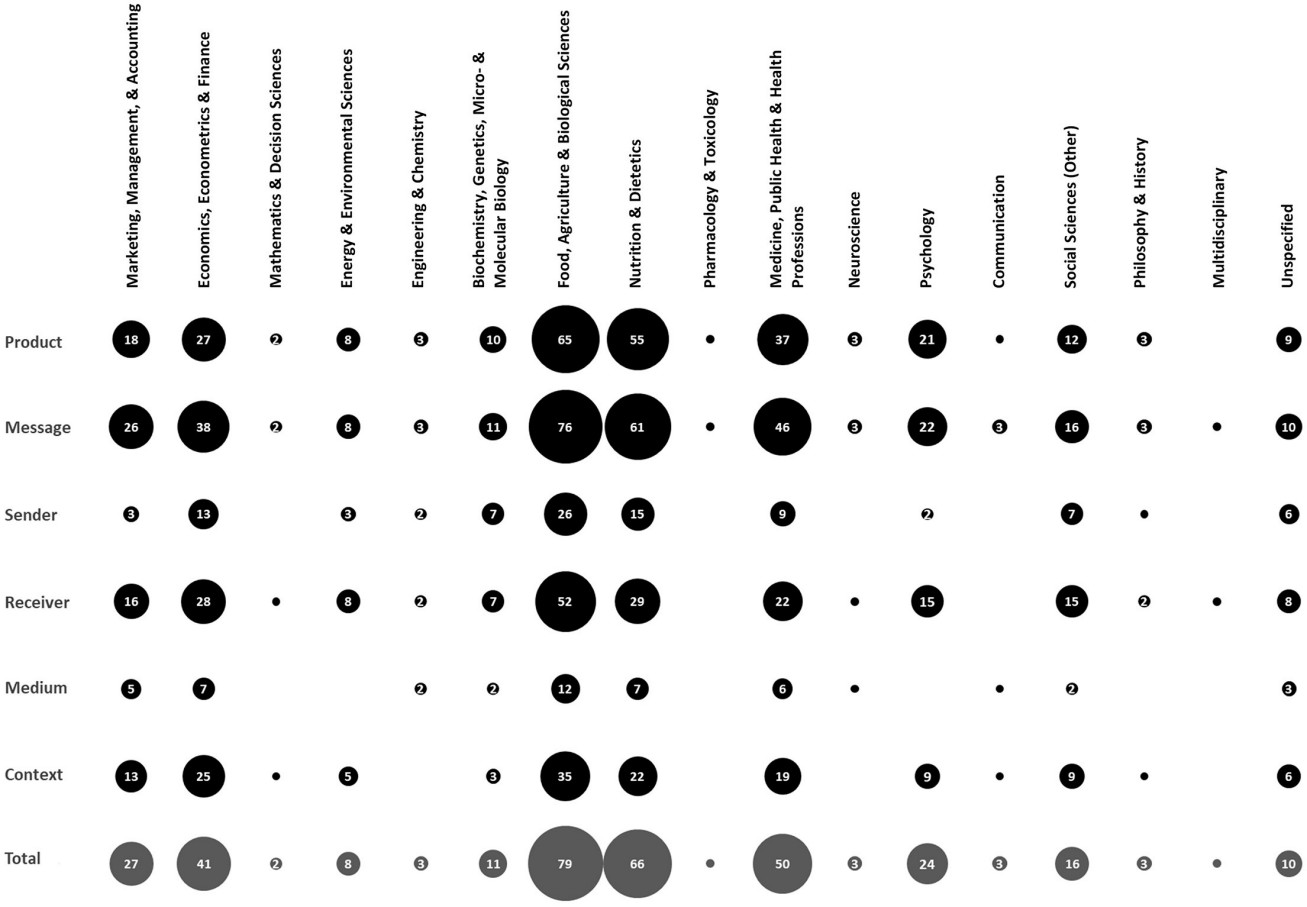
Prevalence of empirical studies in each discipline related to each element (i.e., product, message, sender, receiver, medium, and context) and in total. Numbers in bubbles specify the prevalence. Note that bubbles with no number represent only one record.

Comparing various well-being messaging elements (product, message, sender, receiver, medium, and context), however, indicated that the increase in number of empirical studies has not been consistent across them. Whereas, studies of well-being messaging for milk-investigating product, message, and receiver have all increased over time, studies that focus on sender and medium are now less prevalent, at least in the past 4 years (see Figure 4). Additionally, the number of investigations of each element across time, i.e., *product* (*N* = 141), *message* (*N* = 169), *sender* (*N* = 51), *receiver* (*N* = 101), *medium* (*N* = 27), and *context* (*N* = 72), highlighted a potential gap in knowledge about medium, sender, and even context, compared with the other elements. This knowledge gap seemed to be prevalent across all disciplines interested in milk well-being messaging, albeit with some noticeable discrepancies between disciplines (see Figure 5). Particularly, while product and message have disproportionately been the centres of focus in Nutrition and Dietetics, receiver and, to some extent, context have also received some amount of interest in disciplines such as Food, Agriculture, and Biological Sciences, Medicine, Public Health, and Health Professions, Economic, Econometrics, and Finance, and Marketing, Management, and Accounting.

**Figure 6:**
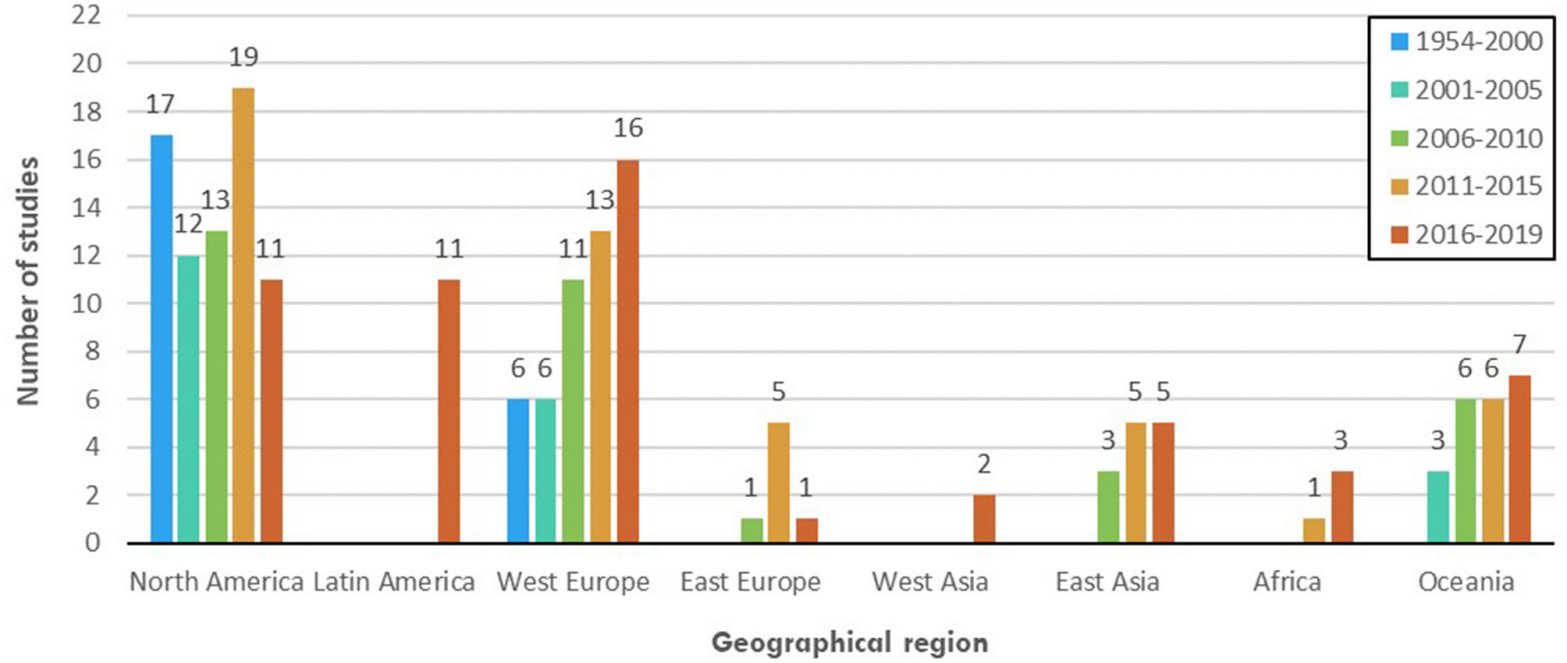
Prevalence of empirical studies in each geographical region (i.e., North America, Latin America, West Europe, East Europe, West Asia, East Asia, and Oceania) during each period (i.e., 1954–2000, 2001–2005, 2006–2010, 2011–2015, and 2016–2019).

**Figure 7:**
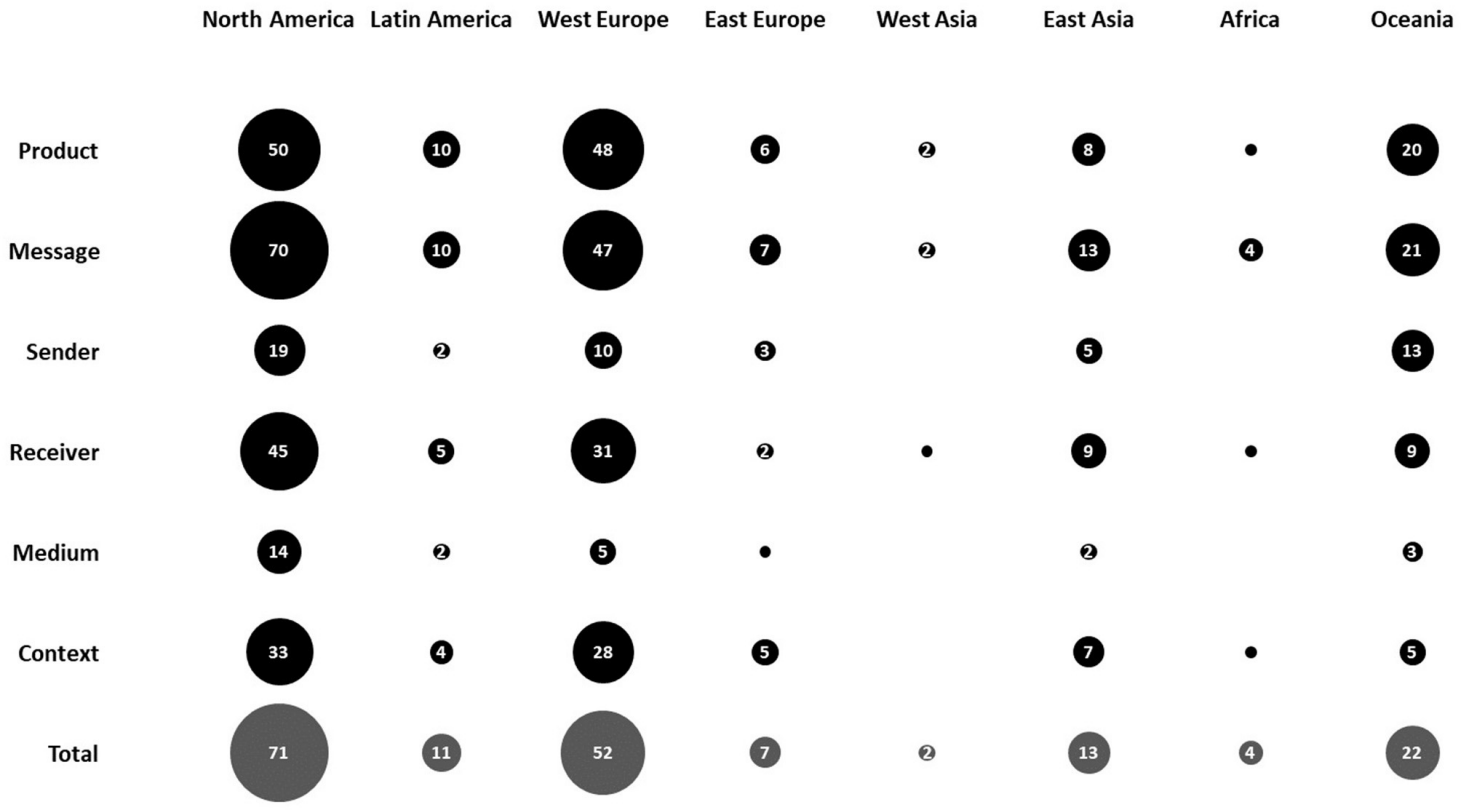
Prevalence of empirical studies in each geographical region (i.e., North America, Latin America, West Europe, East Europe, West Asia, East Asia, and Oceania) related to each element (i.e., product, message, sender, receiver, medium, and context) and in total. Numbers in bubbles specify the prevalence. Note that bubbles with no number represent only one record.

Considering geographical regions, North America, West Europe, and Oceania with 71, 52, and 22 total records, respectively, could be highlighted as hotspots for empirical research in well-being messaging for milk across the time span of this review (see Figure 6). Nevertheless, the past 4 years showed a considerable drop in study numbers in North America. Also, the most recent 10–15 years have witnessed the first few studies emerging in regions such as Latin America, West Asia, and Africa. Most notably, however, East Asia, including populous countries, such as China, India, Indonesia, Pakistan, Bangladesh, Japan, Philippines, and Vietnam, accommodating about half of the World's population, has only been subject to 13 empirical enquiries across time and with no studies published before 2006. With regard to the particular well-being messaging elements of interest, message, and product have been most studied regardless of the geographical region (see Figure 7). Interestingly, context in East Asia and East Europe, two generally less-studied regions, has been included in more than half of the empirical studies of well-being messaging for milk in the region. Also, the receiver of the well-being messaging has been an important element of interest, even more so than product, in studies conducted in East Asia. Finally, sender, while being understudied in general, has featured in 13 out of 22 total studies of well-being messaging for milk in Oceania, considerably more often than in other world regions.

In the following section, a more detailed account of research trends in each element of well-being messaging is presented. In particular, the prevalence of various element-specific aspects in different product types and across time is considered.

#### Well-being Messaging Elements

##### Product

**Figure 8:**
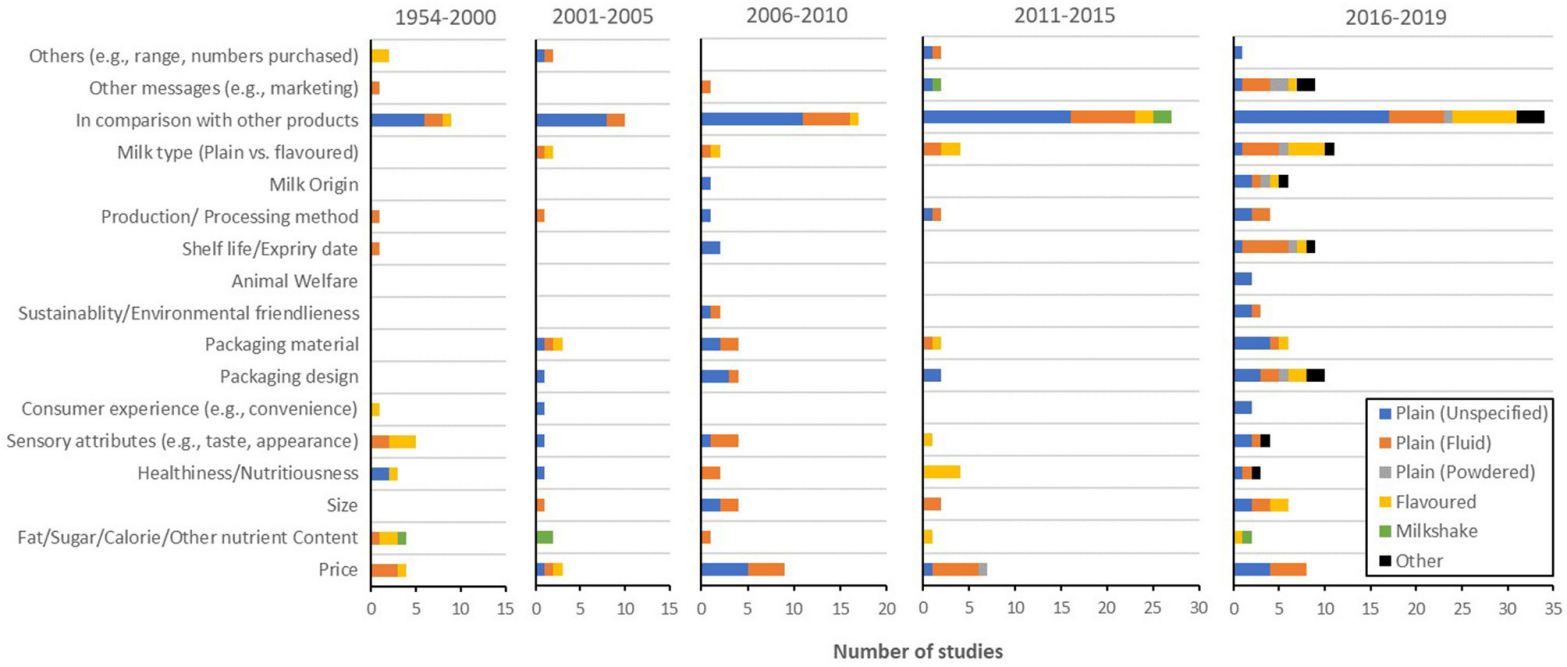
Prevalence of product-related empirical studies for each milk type [i.e., plain (unspecified), plain (fluid), plain (powdered), flavoured milk, milkshake, and other milk drinks] during each period (i.e., 1954–2000, 2001–2005, 2006–2010, 2011–2015, and 2016–2019).

Product was the second most studied well-being message element, after message, across the time span of this review, with recent time periods showing an upward trend in product-related research (Figure 4). A closer look at the overall picture (depicted in Figure 8), however, illustrated that inclusion of products other than milk, providing a point of comparison between products with regard to well-being messaging, was the main driver behind this increase in product-related research. Many other product-related aspects, however, have received less attention across time. For example, whether the product of interest indicated preserving animal welfare and how it might be associated with well-being messaging has mainly been investigated in the most recent time period (2016–2019). This was also the case for milk origin, shelf life or expiry date, and consumer experience. Studies of product-related attributes, such as price, size, sensory attributes, production or processing method, measured healthiness or nutritiousness, and nutrient contents, while being present in most of the time periods, have not played a key part in the increase in number of product-related studies in recent years. Instead, studies focused on packaging (material and design) and comparisons between plain and flavoured milk have increased between 2016 and 2019.

In addition, Figure 8 illustrates a lack of product-related well-being messaging studies on product types other than plain milk (fluid and unspecified). Notably, plain milk powder had never been investigated in the empirical studies of the topic prior to this decade, and research on milkshake has mostly concerned nutrient content.

##### Message

**Figure 9:**
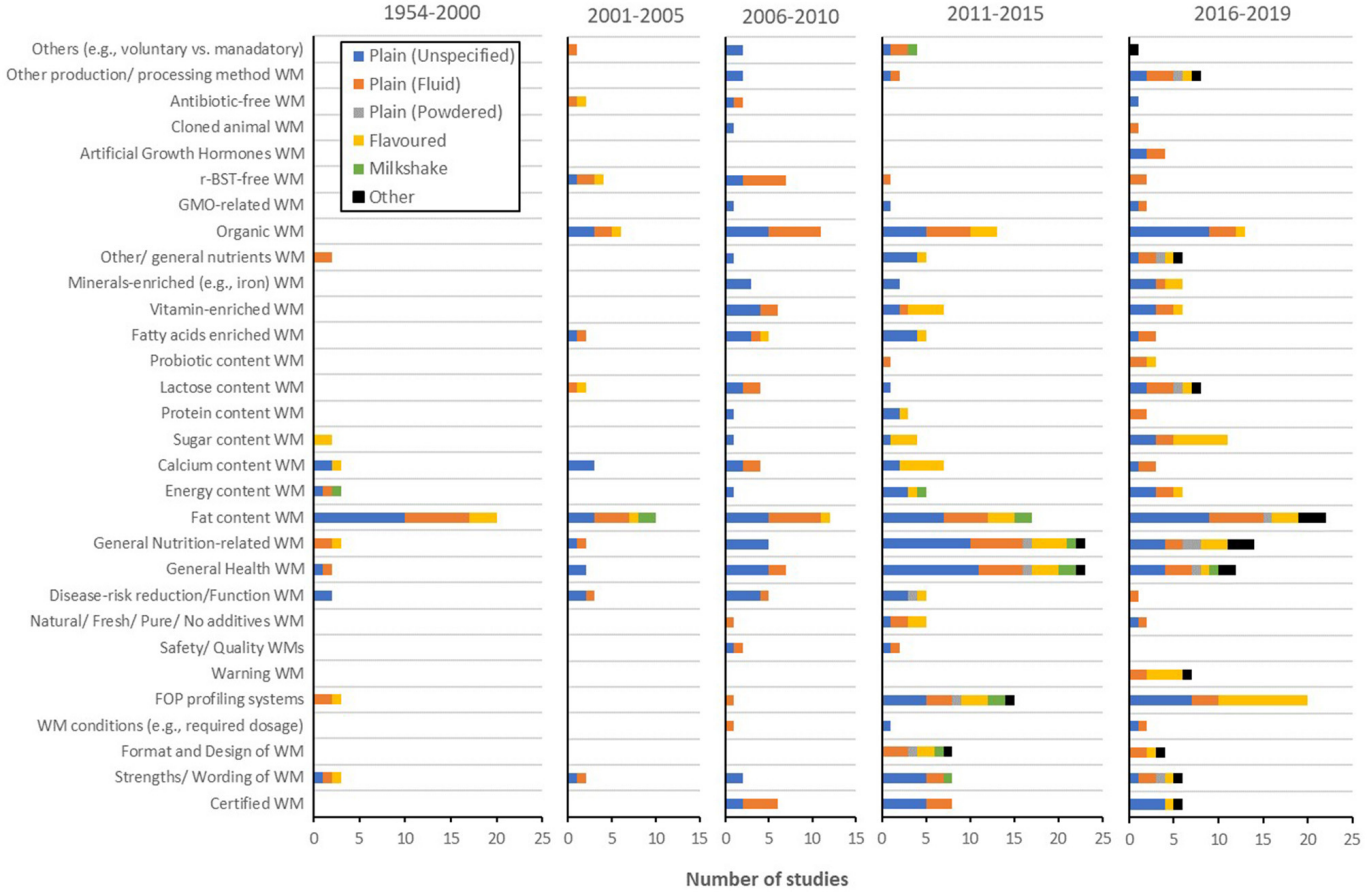
Prevalence of message-related empirical studies for each milk type [i.e., plain (unspecified), plain (fluid), plain (powdered), flavoured milk, milkshake, and other milk drinks] during each period (i.e., 1954–2000, 2001–2005, 2006–2010, 2011–2015, and 2016–2019). WM, well-being messaging; FOP, front-of-pack; r-BST, recombinant bovine somatotropin; GMO, genetically modified.

Message, the most empirically studied well-being messaging element, was arguably the most time-dependent one too. A more detailed precis of message-related studies (Figure 9) showcased the rise and fall of well-being messages carrying various contents across the time. An example of this pattern would be r-BST-free messages (i.e., the cows have not been treated with the hormone recombinant bovine somatotropin). These messages, while not studied prior to 2001, have been the third most commonly empirically examined type of well-being message between 2001 and 2010 (i.e., 10 studies in total). Since 2011, however, only three studies have investigated r-BST-free messages. Another example would be well-being messages around safety or quality of the product that have only been studied between 2006 and 2015 (i.e., four times in total), with no appearance in the records prior to after this time period. On the other hand, well-being messaging on production or processing method, utilisation of artificial growth hormone, genetic modification, lactose, sugar, minerals content, warning messages, the use of front-of-pack (FOP) profiling systems, certified well-being messages, and attention to format and design of well-being messaging have all gained momentum lately. An exception to the time-dependent manner of message-related studies was certainly fat content, which has been an ever-present message across time.

With regard to product type, well-being messaging on sugar content has been, not surprisingly, more prevalent for flavoured milk. Flavoured milk was also a common product type of interest for investigations of FOP profiling systems and warning messages. Much like product-related research, plain milk powder had rarely been subjected to message-related research prior to 2010. The 13 studies published since then have included strengths, wording, format or design of well-being messaging, FOP profiling systems, disease/risk reduction, function, general health, general nutrition-related well-being messaging, fat, lactose, and general nutrients content, and production/processing method well-being messages.

##### Sender

**Figure 10:**
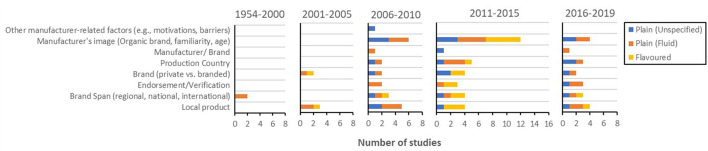
Prevalence of sender-related empirical studies for each milk type [i.e., plain (unspecified), plain (fluid), plain (powdered), flavoured milk, milkshake, and other milk drinks] during each period (i.e., 1954–2000, 2001–2005, 2006–2010, 2011–2015, and 2016–2019).

Sender-related research, while very much lacking till 2006, showed considerable increase across several aspects for the subsequent 10 years. This increase in sender-related studies, however, has not continued in the past four years (Figure 4). Despite the decrease, various sender-related aspects have been investigated (Figure 10). Manufacturer image (e.g., years in business, familiarity to the consumer, and being known as an organic brand) has been frequently studied in relation to well-being messaging lately. Producing country, brand, endorsements or verifications, geographical span of a brand, and whether the product was locally produced or not have all been subjects of empirical enquiry. Manufacturer motivations or barriers to take certain approaches toward well-being messaging, as well as brand, however, were areas of research that have not received much attention across time. A dearth of prior empirical research on this topic was also noticeable with regard to product types other than plain milk (fluid or unspecified) and flavoured milk.

##### Receiver

**Figure 11:**
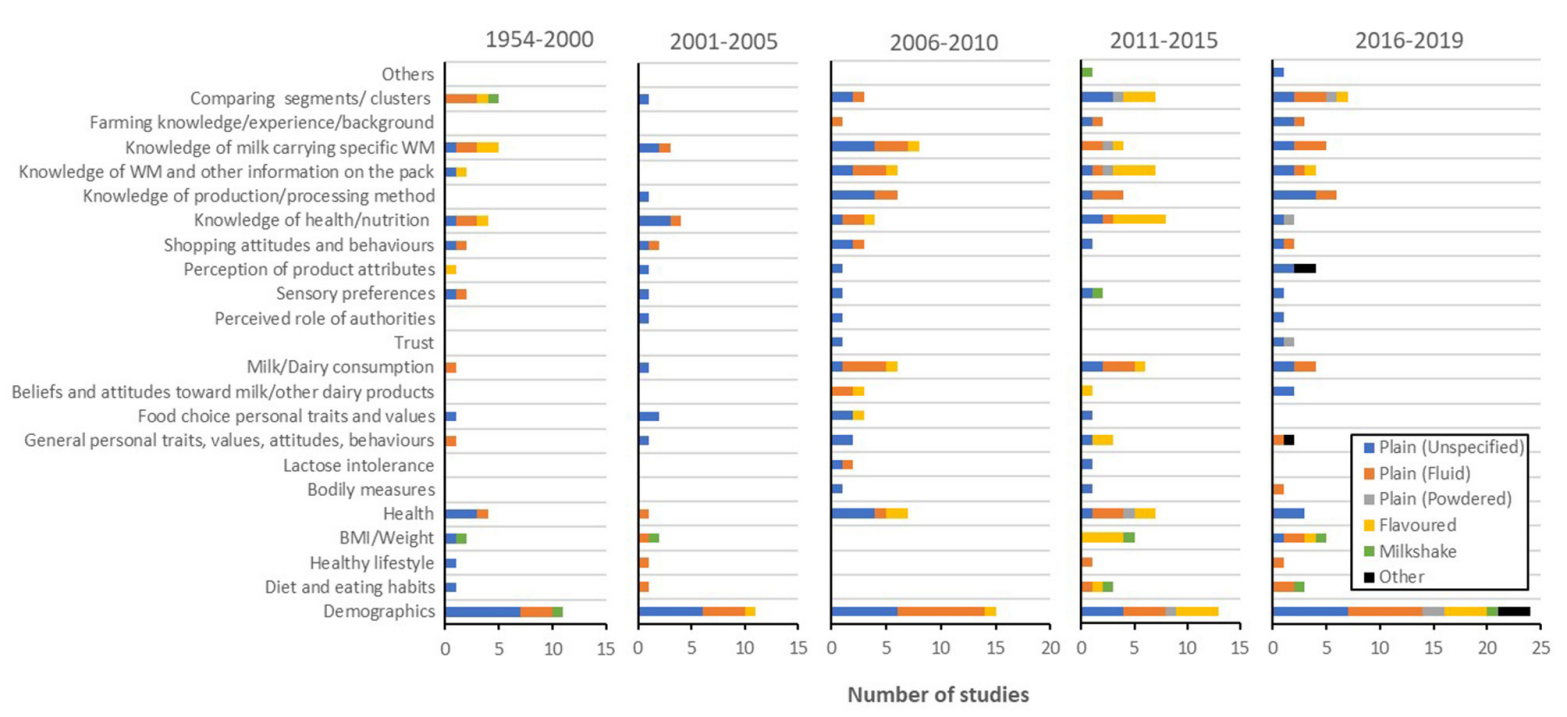
Prevalence of receiver-related empirical studies for each milk type [i.e., plain (unspecified), plain (fluid), plain (powdered), flavoured milk, milkshake, and other milk drinks] during each period (i.e., 1954–2000, 2001–2005, 2006–2010, 2011–2015, and 2016–2019). WM, well-being messaging; BMI, body mass index.

Receiver-related research (Figure 11) quite expectedly included many instances of demographic comparisons (e.g., age, gender, education, and ethnicity). Through time, however, more nuanced receiver-related factors have been included in investigations. Particularly, in the past four years, receiver weight or Body Mass Index (BMI), health, knowledge of production or processing methods, knowledge of specific well-being messages, as well as comparing between various consumer segments or clusters, have each featured at least five times. On the contrary, other factors, such as diet or eating habits, healthy lifestyle, bodily measures, lactose intolerance, individual differences (traits, values, attitudes, or behaviours), food-related individual differences, beliefs and attitudes toward dairy products, sensory preferences, and shopping attitudes and behaviours, while being pivotal to knowing the receiver, have not been subjected to more than 3–10 studies each across the span of the review (i.e., 65 years). Lack of studies on these factors has certainly not been addressed in the past few years. In addition, trust of a receiver in the messages relayed by milk, or a perceived role of authorities, has each only featured in three empirical studies to date.

A lack of studies investigating milk powder as the product of study was a common observation across all elements and was particularly evident here with only 10 studies across the two most recent time periods (2011–2105 and 2016–2019) combined. Comparing these two time periods, it seemed that flavoured milk has also experienced a significant drop in empirical interest lately.

##### Medium

**Figure 12:**
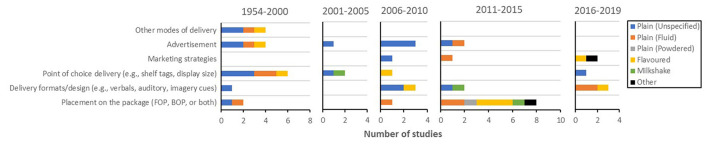
Prevalence of medium-related empirical studies for each milk type [i.e., plain (unspecified), plain (fluid), plain (powdered), flavoured milk, milkshake, and other milk drinks] during each period (i.e., 1954–2000, 2001–2005, 2006–2010, 2011–2015, and 2016–2019). FOP, front-of-pack; BOP, back-of-pack.

Medium was the most clearly overlooked element of well-being messaging research; it has only been included in 27 studies across time, only one-sixth of message-related studies (Figure 4). A closer look at the past four years, however, was more alarming (Figure 12). Placement of well-being messaging on the package (FOP, BOP, or both), for instance, which was studied for various product types between 2011 and 2015, has not been studied at all in this period. Also, research into inclusion of well-being messaging in advertisements, point of choice delivery, and modes of delivery other than the food package has, it would seem, been ignored in recent years. Research on various delivery designs or formats (e.g., verbal, auditory, or imagery cues) and inclusion of marketing strategies in association with well-being messaging for milk, however, has increased lately.

As the number of medium-related studies was quite miniscule, further breaking it down by product type would not provide a meaningful point. Powdered milk, for example, has been studied one time and milkshake three times; then again, the other product types have not been subjected to many more medium-related studies.

##### Context

**Figure 13:**
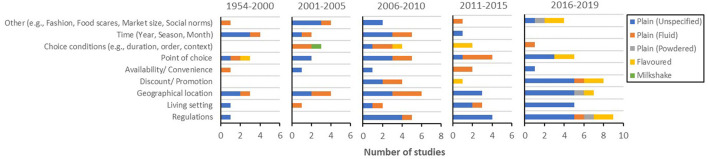
Prevalence of context-related empirical studies for each milk type [i.e., plain (unspecified), plain (fluid), plain (powdered), flavoured milk, milkshake, and other milk drinks] during each period (i.e., 1954–2000, 2001–2005, 2006–2010, 2011–2015), and 2016–2019.

Context-related research in the literature included a variety of factors from point-of-choice-related factors to annual, seasonal, or monthly differences, living conditions, geographical location, and market regulations (Figure 13). While most of these factors have received some attention throughout years, the association between well-being messaging and regulations, geographical location, living settings, point of choice factors, and discounts and/or promotions, among others, have been the main variables of interest in the past four years. In contrast, time, choice conditions, availability, and convenience have not been investigated lately. With regard to less-studied product types and context, whereas milk powder has been studied a few times recently, milkshake was only studied one time back in 2004.

#### Outcome

**Figure 14:**
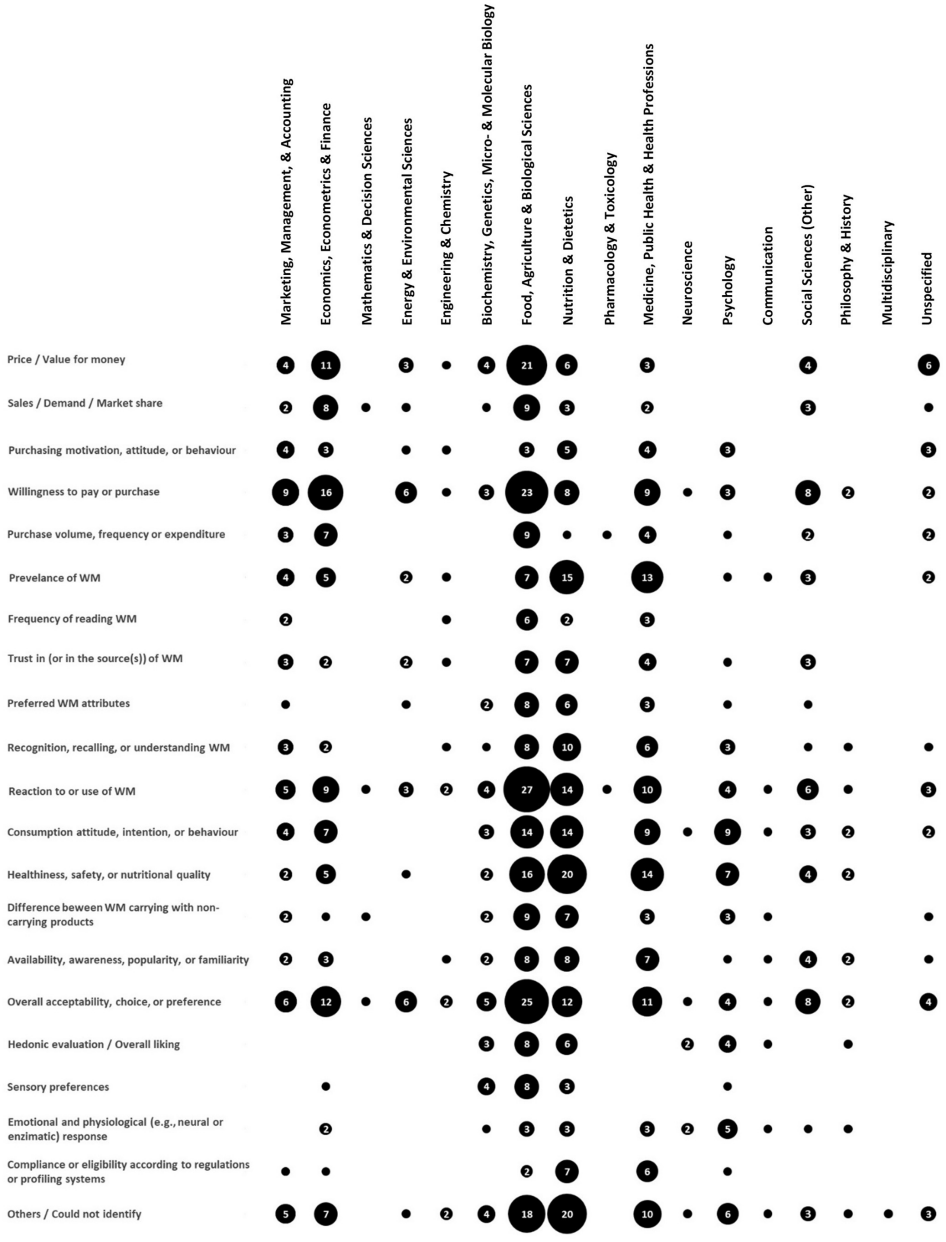
Prevalence of particular outcomes of empirical interest in each discipline. Numbers in bubbles specify the prevalence. Note that bubbles with no number represent only one record.

Well-being messaging for milk, being a multidisciplinary field of research, has attracted a wide range of outcome variables. Figure 14 pictures this variety in association with the scholarly disciplines involved in milk well-being messaging research. Within Food, Agriculture, and Biological Sciences, for instance, price and/or value for money, willingness to pay or purchase, reaction to or use of well-being messaging, overall acceptability, choice, or preference, have each been studied at least 20 times. Interestingly, although, none of these were among the top two outcomes of interest in Nutrition and Dietetics, namely healthiness, safety, or nutritional quality, and prevalence of well-being messaging. Comparing more distant disciplines, the differences between target outcomes became even obvious. Whereas, sales-related outcomes (price and/or value for money, sales, demand, and/or market share, purchasing motivation, attitude, or behaviour, willingness to pay or purchase, and purchase volume, frequency, or expenditure) have been studied 45 times in Economics, Econometrics, and Finance, they have never been subjected to study in Communication. Furthermore, it was surprising to witness the absolute lack of studies targeting hedonic evaluation, overall liking, or sensory preference in Medicine, Public Health, and Health Professions and in Marketing, Management, and Accounting, or price and/or value for money, and sales, demand, and/or market share in Psychology.

## Discussion

An interdisciplinary scoping review of published records on well-being messaging for milk uncovered 246 records, which were analysed from several chronological, geographical, and methodological perspectives, as well as research discipline, product types, and well-being messaging elements of interest. Here, the key findings, strengths, and limitations of this research, and recommendations for future research are discussed.

### Key findings

Eight key findings emerged through this review. First, while as many as 60 review studies were identified in the existing literature, it was surprising to find that milk well-being messaging, *per se*, has not been the main subject of any scoping review to date (see Supplementary Material), and this is despite the considerable number of empirical and nonempirical records that have been published on the topic. Also, given that milk well-being research cuts across various scientific disciplines, a lack of an interdisciplinary approach to reviewing the literature on well-being messaging for milk seems to have played a role in clear discipline-specific knowledge gaps, which are further discussed in this section. Altogether, it is evident that milk well-being research, in its seventh decade of existence, is very much missing scoping reviews aimed at discovering the boundaries of research in the field, clarifying key terms and definitions, and identifying gaps through mapping the literature. This research thus provides a stepping-stone to rectify this limitation, paves the way for more specific research enquiries to bridge the identified gaps, and invites multidisciplinary efforts in milk well-being messaging research.

Second, given that well-being messaging happens in an environment where optimal design, legal compliance, and effective delivery are keys to success, limited research published in disciplines, such as Law or Communication, especially in the past decade, or absolute lack thereof in Arts, Design, or Human Factors and Ergonomics was unexpected observations. Absence of research in these fields may be responsible for largely unheeded well-being messaging elements, such as medium or context. Furthermore, whereas Marketing, Management, and Accounting have published over 30 studies on well-being messaging for milk through the review time span, only three studies have, to date, entertained the sender of well-being messaging. This suggests that future undertakings within the discipline, particularly in areas, such as Organisation Science, Leadership, Process, Innovation, or Strategic Management, are the keys to offer an understanding of the processes of decision-making, design, implementation, and delivery of well-being messaging for milk in organisations.

Third, regarding geographical regions, this review revealed that East Asia, including China and India, nearly half of the World's population, have never been empirically studied before 2006 and have only been subjected to 13 enquiries since then. This is more interesting when considered in conjunction with the number of empirical research studies conducted in Oceania to date (*N* = 22), with a population <45 million. A closer look at the elements of well-being messaging studied across these geographical regions, however, adds further insight. Interestingly, while receiver has been studied in 69.2% of studies carried out in the East Asia region, sender has been investigated in 59.1% of studies conducted in the Oceania region; these are the highest percentages of receiver and sender studies (of total) among all regions. Additionally, when compared with other regions, context in East Asia (53.8% of total studies) and product in Oceania (90.9% of total studies) have been of specific empirical interest. Hence, while East Asia has received meagre studies on milk well-being messaging in general, East Asian receivers and context have been of particular interest. On the other hand, research in Oceania has paid specific attention to the product and the senders of milk well-being messaging. Hence, it might be the case that these findings reflect upon the fact that milk well-being messaging research in East Asia, a major importer of milk (40), and Oceania, a major exporter of milk (40), has responded to the trade market needs for effective well-being messaging *via*: (a) providing a better understanding of consumers and market-related factors in East Asia, and (b) inspecting product- and producer-related aspects in Oceania. Whether scholarly research is ultimately missioned to follow market realities or to provide a comprehensive regime of research investigating all elements of well-being messaging at the same level, however, is a question to be considered collectively by academic society and the dairy industry.

Fourth, milk powder was a subject of only five out of 177 empirical studies of well-being messaging for milk. This figure seems very small when compared to studies of fluid plain milk (55 studies). Considering that most of the 91 studies featuring plain milk of unspecified format are likely to refer to fluid plain milk, the focus on milk powder in plain milk well-being messaging research appears even smaller. Hence, the ratio of well-being messaging research for milk powder to that of plain milk, regardless of its format, is about 1–30. According to Mintel Global New Product Database (41), nearly 1 in 5 plain milks launched in 86 markets around the globe between 2016 and 2019 was milk powder (1,324 out of 6,805 products). In China, for example, 247 of 1,005 plain milks launched in this period were milk powders (24.5%; 39), and, remarkably, none of the five studies that included milk powder in this review were conducted in China. Therefore, there is no doubt that there is a gap in research for well-being messaging concerning powder milk and more so in certain markets.

Fifth, while empirical studies of well-being messaging for milk have been ever-increasing, studies on medium and sender are two elements that have not followed this trend. In fact, the past four years (2016–2019) have shown decreased numbers of empirical studies on medium and sender compared with the two previous periods. While this, as discussed above, might be due to lack of sufficient research in certain disciplines, it might also point to the complexity of conducting research on these elements. Cutting edge research on medium-related (and context-related) aspects of well-being messaging is sometimes reliant on employing novel data collection techniques such as eye-tracking to investigate spatial cognition of product package (42–44) and/or virtual reality to assess consumer interaction with various package designs or point of choice settings (45–47). These methods, while being widespread in other fields of research (48–52), need to become more commonplace in milk well-being messaging research [see (53), for an exception]. Likewise, studies engaging with dairy production, distribution, and retailing functions to understand their motivations, barriers, and procedures regarding design, implementation, and evaluation of well-being messaging [e.g., (54)], despite being more challenging than convenience sampling or collecting on-the-pack information, are vital to sender-related research. Employing lessons learnt from organisation research on other topics such as new product development (52, 55, 56) or food packaging (57) can be helpful.

Sixth, more commonly studied elements, namely message, product, and receiver, have all witnessed consistent increase in scholarly attention during the past two decades. The increase in number of studies, however, does not necessarily mean that our understanding of aspects of well-being messaging related to these elements is also growing comprehensively. Research on product, for instance, has disproportionally involved the inclusion of food products other than milk as points of comparison. Many other important product-related aspects, such as milk origin, shelf life, packaging (material and design), sensory attributes, consumer experience, size, production or processing method, measured healthiness or nutritiousness, and nutrient content, however, have arguably received insufficient attention across time. This is also the case for receiver-related research, where inclusion of demographic information (e.g., age, gender, education, and ethnicity) has been and increasingly has become the most common practise. This is despite the existence of many understudied aspects, such as consumer diet, eating habits, healthy lifestyle, bodily measures, lactose intolerance, general and food-related individual differences (traits, values, attitudes, or behaviours), beliefs and attitudes toward dairy products, sensory preferences, and shopping attitudes and behaviours that are crucial pieces of the puzzle. In regard to message-related research, however, at least, during the past four years (2016–2019), various message types, formats, designs, and content have all been subjected to empirical research.

Seventh, a closer look at message-related research reveals a time-dependent pattern in empirical studies of particular well-being messages for milk. As such, antibiotic-free or r-BST-free messages or well-being messages around safety or quality of the product were all more common between 2001 and 2015, compared with other times periods. This might be explained through short-lived food trends or food scares during this period. A search in Google Trends archival data (performed on 9th March 2021), for instance, shows that worldwide Google searches were substantially more common for the terms “rBST in milk” prior to 2010 and “antibiotic in milk” prior to 2008, compared with years after. This showcases a significant increase in public attention to these topics, which might have been triggered by other regional or global events at the time, possibly convincing the dairy industry to consider relevant well-being messaging, and ultimately encouraging higher scientific attention to the topic. The infamous case of melamine adulteration in milk and infant formula in 2008, which resulted in death and hospitalisation of babies and adults across China (58), for instance, caused a peak in scholarly interest in safety and quality well-being messaging for a short period thereafter. Whether other emerging trends, such as messages on production/processing method, such as cloned animals, artificial growth hormones, genetically modified, and organic, or messages on lactose, probiotic, vitamins, and minerals content, will stand the test of time, however, needs to be reassessed in future reviews of this topic. Having said that, there are aspects, including fat, calcium, and sugar content; strengths and wording of well-being messages; and well-being messaging on health and nutrition values of milk, that have generally remained mainstream in message-related research.

Eighth, whereas wide-ranging outcome variables have been of interest in milk well-being messaging research, this research highlights that various disciplines have focussed on specific outcomes and, sometimes, to the extent that they have missed other important ones. A lack of interdisciplinary reviews of the topic has not helped this situation either. Discipline-specific tunnel vision is clear in several areas, for example, lack of consideration of price, value for money, sales, demand, and market share in research published in Psychology or Communication disciplines; or the omission of hedonic evaluation, overall liking, and sensory preference factors in Medicine, Public Health, and Health Professions discipline, and in Marketing, Management, and Accounting research. Hence, taking an interdisciplinary lens would result in a more complete depiction of milk well-being messaging, one that is more capable of guiding optimal well-being messaging policies and practises.

### Strengths and Limitations

The research was crucially founded on three proposed shifts in well-being messaging research paradigms. These, on their own, can be considered as important contributions to the topic. First is the use of “well-being,” as a multifaceted concept covering various biopsychosocial domains instead of “health,” which is commonly understood as the state of being physically healthy. Also, “well-being messaging” has been suggested as an umbrella term that contains various types, frames, designs, contents, and strengths of well-being-related food labels appearing on- and/or off-package. Adopting these terms and definitions was the key to bringing together a seemingly diverging literature around what is essentially *well-being messaging*. Second, well-being messaging was modelled as an act of communication and, hence, consisted of distinct communication elements. This novel approach allowed the identification of aspects of milk well-being messaging that have not received much attention in the past, e.g., sender and medium. Third, well-being messaging, particularly when broadly defined, lends itself to various research enquiries that, in many cases, cuts across various scientific disciplines. Taking an interdisciplinary approach to reviewing milk well-being messaging research showcased discipline-specific vision that caused some disciplines to focus on specific outcomes while missing important ones. Following this approach in future scholarly research will provide more comprehensive and informative research on milk well-being messaging.

However, there are also a few limitations to note. First, given the overwhelming number of identified records, and in line with the recommendations for conducting scoping reviews, quality appraisal was not performed on identified records. Second, in order to maintain the feasibility of this scoping review, eligibility criteria were used to philtre out records with no access to full texts through institutional holdings available to the authors, potential grey literature indexed in other data sources that also did not appear in cited in-text or in reference lists of the general well-being messaging database records, non-English records, and records in which findings exclusive to milk or other product types of interest were not separated from other products (e.g., dairy products, milk, and plant-based milk substitutes). Third, while taking an interdisciplinary approach to conducting this scoping review allowed an open approach to emerging themes and to accommodate wide-ranging designs, variables, and outcomes of interest, this approach also limited the possibility for the fine-grained data synthesis a systematic review with a narrower aperture would allow.

## Conclusion

This research is the first interdisciplinary scoping review of well-being messaging for milk. It features unique aspects, including: (i) offering an all-embracing definition of well-being messaging; (ii) suggesting a framing model for well-being messaging research; (iii) taking an interdisciplinary approach to the search, synthesis, and analysis of literature; (iv) inclusion of both empirical and non-empirical records; (v) providing a comprehensive overview of milk well-being messaging research (across scientific disciplines, time, geographical locations, product type, and research methods) as well as a closer look at particular knowledge gaps that require further attention. Most importantly, existing non-empirical literature to date has not included an exclusive review of research on well-being messaging for milk. The results of this review also highlight notable knowledge gaps in the empirical literature, particularly with regard to milk well-being messaging research in geographical locations other than North America, West Europe, and Oceania, on product types other than fluid plain milk, and on messaging elements other than product and message. Insights from this research not only warrant the need for further academic undertakings in certain domains of milk well-being messaging but also inform practitioners and policy-makers who can understand chronological well-being needs and concerns of milk consumers in various markets as reflected in areas of research focus across time.

## Author Contributions

SM and JH designed the study, screened the results, and wrote the paper. SM conducted the literature search and analysed and charted the results. All authors contributed to the article and approved the submitted version.

## Funding

This research was funded by the New Zealand Ministry of Business, Innovation and Employment (MBIE) through the New Zealand Milks Mean More (NZ3M) Endeavour Programme (Contract MAUX1803). The New Zealand Milks Mean More (NZ3M) Endeavour Programme funded by the Ministry of Business, Innovation and Employment (MBIE) is a collaborative partnership between the Riddet Institute, AgResearch Ltd, the University of Auckland and the University of Otago. The Programme is hosted and led by the Riddet Institute at Massey University.

## Conflict of Interest

The authors declare that the research was conducted in the absence of any commercial or financial relationships that could be construed as a potential conflict of interest.

## Publisher's Note

All claims expressed in this article are solely those of the authors and do not necessarily represent those of their affiliated organizations, or those of the publisher, the editors and the reviewers. Any product that may be evaluated in this article, or claim that may be made by its manufacturer, is not guaranteed or endorsed by the publisher.

## References

[1] Scholz-Ahrens KE Ahrens F Barth CA. Nutritional and health attributes of milk and milk imitations. Eur J Nutr. (2020) 59:19–34. 10.1007/s00394-019-01936-3 30937581

[2] Pereira PC. Milk nutritional composition and its role in human health. Nutrition. (2014) 30:619–27. 10.1016/j.nut.2013.10.011 24800664

[3] Thorning TK Raben A Tholstrup T Soedamah-Muthu SS Givens I Astrup A. Milk and dairy products: good or bad for human health? An assessment of the totality of scientific evidence. Food Nutr Res. (2016) 60:32527. 10.3402/fnr.v60.32527 27882862 PMC5122229

[4] Willett WC Ludwig DS. Milk and health. N Engl J Med. (2020) 382:644–54. 10.1056/NEJMra1903547 32053300

[5] Gómez-Cortés P Juárez M de la Fuente MA. Milk fatty acids and potential health benefits: an updated vision. Trends Food Sci Tech. (2018) 81:1–9. 10.1016/j.tifs.2018.08.014

[6] Visioli F Strata A. Milk, dairy products, and their functional effects in humans: a narrative review of recent evidence. Adv Nutr. (2014) 5:131–43. 10.3945/an.113.005025 24618755 PMC3951796

[7] von Philipsborn P Stratil JM Burns J Busert LK Pfadenhauer LM Polus S. Environmental interventions to reduce the consumption of sugar-sweetened beverages and their effects on health. Cochrane Db Syst Rev. (2019) 6:CD012292. 10.1002/14651858.CD012292.pub2 31194900 PMC6564085

[8] Brown HM Rollo ME de Vlieger NM Collins CE Bucher T. Influence of the nutrition and health information presented on food labels on portion size consumed: a systematic review. Nutr Rev. (2018) 76:655–77. 10.1093/nutrit/nuy019 29767760

[9] Kurajdova KT-P Janka. Literature review on factors influencing milk purchase behaviour. Int Rev Manag Mark. (2015) 5:9–25.

[10] Leathwood PD Richardson DP Strater P Todd PM van Trijp HC. Consumer understanding of nutrition and health claims: sources of evidence. Br J Nutr. (2007) 98:474–84. 10.1017/S000711450778697X 17705892

[11] Dolgopolova I Teuber R. Consumers' willingness to pay for health benefits in food products: a meta-analysis. Appl Econ Perspect P. (2018) 40:333–52. 10.1093/aepp/ppx036

[12] Anastasiou K Miller M Dickinson K. The relationship between food label use and dietary intake in adults: a systematic review. Appetite. (2019) 138:280–91. 10.1016/j.appet.2019.03.025 31010704

[13] Shangguan S Afshin A Shulkin M Ma W Marsden D Smith J. A meta-analysis of food labeling effects on consumer diet behaviors and industry practices. Am J Prev Med. (2019) 56:300–14. 10.1016/j.amepre.2018.09.024 30573335 PMC6340779

[14] Campos S Doxey J Hammond D. Nutrition labels on pre-packaged foods: a systematic review. Public Health Nutr. (2011) 14:1496–506. 10.1017/S1368980010003290 21241532

[15] Steinhauser J Hamm U. Consumer and product-specific characteristics influencing the effect of nutrition, health and risk reduction claims on preferences and purchase behavior - a systematic review. Appetite. (2018) 127:303–23. 10.1016/j.appet.2018.05.012 29772293

[16] Hawley KL Roberto CA Bragg MA Liu PJ Schwartz MB Brownell KD. The science on front-of-package food labels. Public Health Nutr. (2013) 16:430–9. 10.1017/S1368980012000754 22440538 PMC10271311

[17] Moradi S Van Quaquebeke N Hunter JA. Flourishing and prosocial behaviors: a multilevel investigation of national corruption level as a moderator. PLoS ONE. (2018) 13:e91473. 10.1371/journal.pone.0200062 30001332 PMC6042718

[18] Dodge R Daly AP Huyton J Sanders LD. The challenge of defining wellbeing. Int J Wellbeing. (2012) 3:222–35. 10.5502/ijw.v2i3.4

[19] Cloninger CR. Feeling Good: The Science of Well-Being. New York, NY: Oxford University Press (2004).

[20] Aguirre P. The culture of milk in Argentina. Anthropol J Food. (2003) 2:1–12. 10.4000/aof.322

[21] Hakkarainen R Partonen T Haukka J Virtamo J Albanes D Lönnqvist J. Food and nutrient intake in relation to mental wellbeing. Nutr J. (2004) 3:14. 10.1186/1475-2891-3-14 15363099 PMC519023

[22] Hong SA Peltzer K. Dietary behaviour, psychological well-being and mental distress among adolescents in Korea. Child Adol Psych Men. (2017) 11:56. 10.1186/s13034-017-0194-z 29209411 PMC5706161

[23] Yamamura S Morishima H Kumano-Go T Suganuma N Matsumoto H Adachi H. The effect of *Lactobacillus helveticus* fermented milk on sleep and health perception in elderly subjects. Europ J Clin Nutr. (2009) 63:100–5. 10.1038/sj.ejcn.1602898 17851460

[24] Lähteenmäki L Lahteenmaki L Lähteenmäki L. Claiming health in food products. Food Qual Prefer. (2013) 27:196–201. 10.1016/j.foodqual.2012.03.006

[25] Grant MJ Booth A. A typology of reviews: an analysis of 14 review types and associated methodologies. Health Info Libr J. (2009) 26:91–108. 10.1111/j.1471-1842.2009.00848.x 19490148

[26] EPPI-Centre. EPPI-Centre Methods for Conducting Systematic Reviews. London: EPPI Centre, Social Science Research unit, Institute of Education, University of London (2006).

[27] Carroll C Booth A Cooper K. A worked example of “best fit” framework synthesis: a systematic review of views concerning the taking of some potential chemopreventive agents. BMC Med Res Methodol. (2011) 11:29. 10.1186/1471-2288-11-29 21410933 PMC3068987

[28] Lasswell HD. The structure and function of communication in society. In: Bryan L editor. The Communication of Ideas: A Series of Addresses. New York, NY: Harper & Bros (1948). p. 215–28.

[29] Eunson B. Communicating in the 21st Century. Milton, QLD: John Wiley & Sons Australia, Limited (2015).

[30] Tricco AC Lillie E Zarin W O'Brien KK Colquhoun H Levac D. PRISMA extension for scoping reviews (PRISMA-ScR): checklist and explanation. Ann Intern Med. (2018) 169:467–73. 10.7326/M18-0850 30178033

[31] Pham MT Rajić A Greig JD Sargeant JM Papadopoulos A McEwen SA. A scoping review of scoping reviews: advancing the approach and enhancing the consistency. Res Synth Methods. (2014) 5:371–85. 10.1002/jrsm.1123 26052958 PMC4491356

[32] Arksey H O'Malley L. Scoping studies: towards a methodological framework. Int J Soc Res Method. (2005) 8:19–32. 10.1080/1364557032000119616

[33] Levac D Colquhoun H O'Brien KK. Scoping studies: advancing the methodology. Implement Sci. (2010) 5:69. 10.1186/1748-5908-5-69 20854677 PMC2954944

[34] Tricco AC Lillie E Zarin W O'Brien K Colquhoun H Kastner M. A scoping review on the conduct and reporting of scoping reviews. BMC Med Res Methodol. (2016) 16:15. 10.1186/s12874-016-0116-4 26857112 PMC4746911

[35] Petersen P Feldt R Mujtaba S Mattsson M. Systematic mapping studies in software engineering. In: Proceedings of the 12th International Conference on Evaluation and Assessment in Software Engineering. Italy: BCS Learning &amp; Development Ltd. (2008). p. 68–77. 10.14236/ewic/EASE2008.8

[36] Petersen K Vakkalanka S Kuzniarz L. Guidelines for conducting systematic mapping studies in software engineering: an update. Inform Software Tech. (2015) 64:1–18. 10.1016/j.infsof.2015.03.007

[37] SIM Review Protocol-Revised,.pdf (Version: 1)-Open Science Framework Repository (2021). Available online at: https://osf.io/extr2/?view_only=ae1588e3a8a54e688c4021161684b0e2 (accessed August 3, 2021)

[38] Ouzzani M Hammady H Fedorowicz Z Elmagarmid A. Rayyan—a web and mobile app for systematic reviews. Syst Rev. (2016) 5:210. 10.1186/s13643-016-0384-4 27919275 PMC5139140

[39] World Map Region Definitions-Our World in Data. (2021). Available online at: https://ourworldindata.org/world-region-map-definitions (accessed March 18, 2021)

[40] Food and Agriculture Organisation. OECD-FAO Agricultural Outlook 2020-2029. Paris: Food & Agriculture Organisation (2020).

[41] Mintel GNPD - Global New Products Database: CPG FMC | Mintel.com (2021). Available online at: https://www.mintel.com/global-new-products-database (accessed March 18, 2021).

[42] Kim M-A Yoo H-J Ares G Lee H-S. Effect of thinking style and consumption purpose on food choice: a case study with yogurt using a discrete choice experiment and eye-tracking. Food Qual Prefer. (2020) 86:104025. 10.1016/j.foodqual.2020.104025

[43] Ploom K Kuusik A Pentus K Mehine T Koiv M Tempel A. Mobile and stationary eye tracking comparison – package design and in-store results. J Consum Mark. (2020) 37:259–69. 10.1108/JCM-04-2019-3190

[44] Piqueras-Fiszman B Velasco C Salgado-Montejo A Spence C. Using combined eye tracking and word association in order to assess novel packaging solutions: a case study involving jam jars. Food Qual Prefer. (2013) 28:328–38. 10.1016/j.foodqual.2012.10.006

[45] Blom SSAH Gillebaart M De Boer F van der Laan N De Ridder DTD. Under pressure: nudging increases healthy food choice in a virtual reality supermarket, irrespective of system 1 reasoning. Appetite. (2021) 160:105116. 10.1016/j.appet.2021.105116 33450297

[46] Siegrist M Ung C-Y Zank M Marinello M Kunz A Hartmann C. Consumers' food selection behaviors in three-dimensional (3D) virtual reality. Food Res Int. (2019) 117:50–9. 10.1016/j.foodres.2018.02.033 30736923

[47] Ruppert B. New directions in the use of virtual reality for food shopping: marketing and education perspectives. J Diabetes Sci Technol. (2011) 5:315–8. 10.1177/193229681100500217 21527099 PMC3125922

[48] Lai M-L Tsai M-J Yang F-Y Hsu C-Y Liu T-C Lee SW-Y. A review of using eye-tracking technology in exploring learning from 2000 to 2012. Edu Res Rev-Neth. (2013) 10:90–115. 10.1016/j.edurev.2013.10.001

[49] Scott N Zhang R Le D Moyle B. A review of eye-tracking research in tourism. Curr Issues Tour. (2019) 22:1244–61. 10.1080/13683500.2017.1367367

[50] Laver K George S Thomas S Deutsch JE Crotty M. Cochrane review: virtual reality for stroke rehabilitation. Eur J Phys Rehabil Med. (2012) 48:523–30. 10.1002/14651858.CD008349.pub2 22713539

[51] Diemer J Alpers GW Peperkorn HM Shiban Y Mühlberger A. The impact of perception and presence on emotional reactions: a review of research in virtual reality. Front Psychol. (2015) 6:26. 10.3389/fpsyg.2015.00026 25688218 PMC4311610

[52] Seth A Vance JM Oliver JH. Virtual reality for assembly methods prototyping: a review. Virtual Real-London. (2011) 15:5–20. 10.1007/s10055-009-0153-y

[53] Oliveira D Machin L Deliza R Rosenthal A Walter EH Gimenez A. Consumers' attention to functional food labels: Insights from eye-tracking and change detection in a case study with probiotic milk. Lwt-Food Sci Technol. (2016) 68:160–7. 10.1016/j.lwt.2015.11.066

[54] Stojanović ŽZ-L. Martine: Esteve, Magali. Study Report on Consumer Motivations and Behaviours for Products with Health Claims. BEL-Faculty of Economics-University of Belgrade (2010).

[55] Johnson JS Friend SB Lee HS. Big Data facilitation, utilization, and monetization: exploring the 3Vs in a new product development process. J Prod Innovat Manag. (2017) 34:640–58. 10.1111/jpim.12397

[56] Tiedemann F Johansson E Gosling J. Structuring a new product development process portfolio using decoupling thinking. Prod Plan Control. (2020) 31:38–59. 10.1080/09537287.2019.1629037

[57] Simms CD Trott P. The Dysfunctional Nature of Packaging Development: An Exploratory Study in the UK Food Industry. Copenhagen: DRUID Society; CBS (2014).

[58] Xiu C Klein KK. Melamine in milk products in China: examining the factors that led to deliberate use of the contaminant. Food Policy. (2010) 35:463–70. 10.1016/j.foodpol.2010.05.001

