# Supplementary material for: Well-being Messaging for Mammalian Milks: A Scoping Review
Source: Front Nutr. 2021 Oct 22;8:688739. doi: 10.3389/fnut.2021.688739 (PMC8570841; doi:10.3389/fnut.2021.688739)
Supplement: Supplementary file 1 [file Data_Sheet_1.docx]

**Supplementary Material**


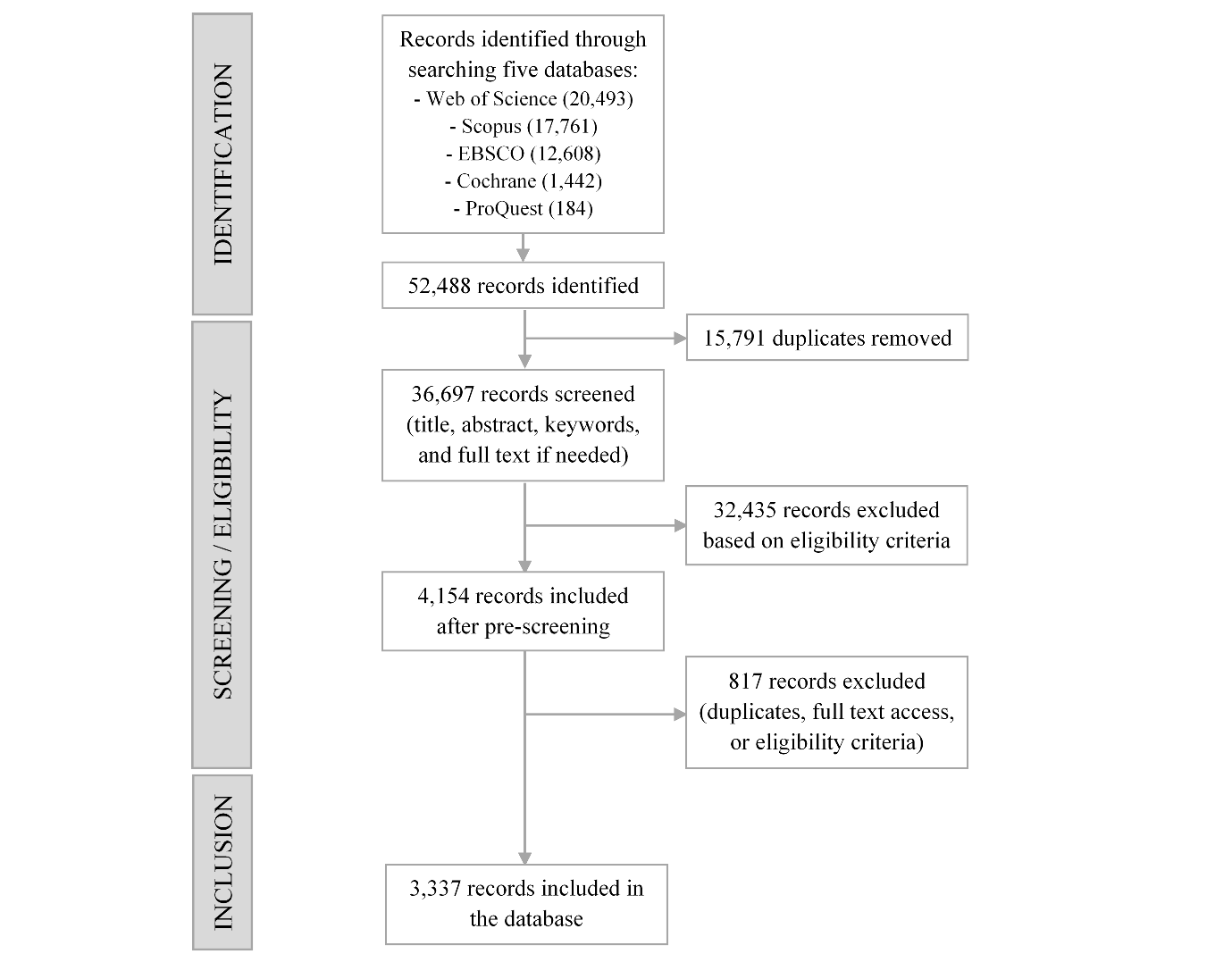


Figure 1: Search flowchart resulting in the well-being messaging database

Table 1: A priori coding framework for charting the data

| General categories | Empirical-exclusive categories | Non-empirical exclusive categories |
| --- | --- | --- |
| Year of publication | Research method | Publication type |
| Publication outlet | Study design^*^ | Brief summary of content |
| Scientific discipline(s) | Product type |  |
| Record type | Country(-ies) of study |  |
|  | Well-being messaging elements of focus |  |
|  | Outcomes of interest |  |

^*^Study design was later dropped from the coding framework.

Table 2: A summary of all non-empirical records (reviews, meta-analysis, case reports, conceptual pieces, and opinion papers) included in the review sorted by publication year (descending order) and first author name (alphabetical order).

| (1) | (2) | (3) | (4) | (5) |
| --- | --- | --- | --- | --- |
| A list of commercial milk across the world as a functional food with health benefits based on bioactive peptides/protein hydrolysates and relavant health claim regulations in different countries (China, Japan, European Union, United States, Canada). **(Review) (Journal Article)** | The effects of environmental interventions (e.g., emoticon labelling, incentivizing the selection of healthier milk, and placement of milk in school cafeterias, and improved access to drinking water) on the consumption of sugar-sweetened milk, diet-related anthropometric measures and health outcomes, and on any reported unintended consequences or adverse outcomes. (Review) (Journal Article) | Influence of the nutrition and health information (e.g., fat content, energy content) presented on food labels on portion size consumed. (Review) (Journal Article) | A meta-review of the effectiveness of health claim (i.e., bone health, diabetes) on marginal willingness to pay for milk in comparison to other food products. (Review) (Journal Article) | Examples of meal replacement milkshakes currently available in the UK market and their advertised nutrition and/or health claims. (Review) (Book Chapter) |
| (6) | (7) | (8) | (9) | (10) |
| Review of studies reporting product and consumer-specific characteristics influencing the effect of nutrition, health, risk-reduction claims. **(Review) (Journal Article)** | Regulations regarding commercial use and labeling of rBST-treated milk sources, rBST-free and organic labeled milk. (Review) (Book Chapter) | French regulations regarding labelling of the milk origin. (Case Report) (Journal Article) | Code of Federal Regulations and Codex Alimentarius Standards, Australia and New Zealand, and Japan Regulations regarding well-being messaging, particularly skim or nonfat milk labelling, nutritional labelling, and nutritional content claims (e.g., protein, calcium, iodine, phosphorous). (Review) (Book Chapter) | Comparison between Canadian, US, and FAO proposed regulatory systems, particularly the claim statements permissible under various regulatory systems (e.g., protein content claim statements for whole milk). (Review) (Journal Article) |
| (11) | (12) | (13) | (14) | (15) |
| Provides a brief introduction to the regulations pertaining to milk labeling in Pakistan (e.g., language of the information on the label should include both Urdu and English, ruminant's name from which milk is derived should be declared, and skim milk should be clearly labeled as "Not Fit for Babies"). **(Review) (Journal Article)** | FDA regulation regarding the labelling of the trans fatty acids in milk. (Review) (Journal Article) | The Ohio Department of Agriculture and FDA regulations to address the "r-BST free" cow milk. (Review) (Journal Article) | A list of commercial milk drinks fortified with calcium, omega-3, soy isoflavones, and protein targeting a particular health trend (i.e., bone health, menopausal effects, performance) and market segments (e.g., young professionals, middle-aged women). “Got milk?” campaign’s effect on consumer awareness of calcium importance for bone and teeth health. Consumer perception of milk as functional food, and milk as the best source of calcium Success of milk fortified with calcium and omega-3 as functional food. (Review) (Book Chapter) | A list of commercial milk as a functional food with supplemented elements and health claims (e.g., ProDiet F200, Reduces stress). (Review) (Book Chapter) |
| (16) | (17) | (18) | (19) | (20) |
| Provides a review of the factors influencing milk purchase behaviour including well-being messaging. **(Review) (Journal Article)** | Reviews the evolution of Thai food and nutrition label policies, with specific attention to milk labeling regulations about declaring the 'type of food' and 'place of production' for many types of products such as condensed milk, milk powder, and colostrum. Also, discusses the impact of Thailand regulations on regulations developed in other countries in the region (e.g., Vietnam). (Review) (Journal Article) | A list of commercial milk across the world as a functional food with active compounds. (Review) (Journal Article) | Review of genetically engineered milk labeling according to the Vermont Genetically Engineered Food Labeling Act. (Review) (Journal Article) | A case report in the Court of Justice of the European Union that includes brief legal arguments regarding milk being a product with beneficial health effects. (Case Report) (Journal Article) |
| (21) | (22) | (23) | (24) | (25) |
| Qualified well-being messaging according to Japanese regulations. **(Review) (Book Chapter)** | A brief review of milk approved as functional food across Asian legislations, including "phytosterol-enriched milk powder for heart health" by the Indonesian National Agency for Drug & Food Control, or a sterol-containing milk drink approved to bear a label claim ‘An animal study shows that consumption of this product may help lower blood total cholesterol’ by the Taiwanese Health Food Control Act", and a detailed verbatim analysis of its label information. Plus, "Nestle Functional milk enriched with calcium, omega-3 and omega-6 fatty acids, and plant sterols" compliance with Singaporean legislations. (Review) (Book Chapter) | Review of literature on safety perception of food products, including “green” liquid milk, among Chinese consumers. (Review) (Journal Article) | Provides a discussion on fluid milk being the most common product with fat claims in USA in 1997. (Review) (Conference Paper) | Provides an overview of empirical evidence on the effectiveness of product health information for food products, including milk, at the point of purchase. (Review) (Journal Article) |
| (26) | (27) | (28) | (29) | (30) |
| Health claim for drinkable milk permissible according to Bosnia and Herzegovina’s regulations. **(Review) (Conference Paper)** | A discussion of rBST-free label as an instance of "Interattribute Misleadings" in case it causes misbelief of milk being safer and/or higher quality than milk from rBST-treated cows. (Conceptual Piece) (Journal Article) | Various types, forms, and contents of well-being messaging as well as other information communicable via milk to the consumer have been reviewed from the Codex Alimentarius regulation point of view. (Review) (Book Chapter) | A list of commercial functional dairy products (including milk drinks) fortified with beneficial ingredient and the manufacturer. (Review) (Journal Article) | Review of milk as functional food carrying bioactive components (e.g., CLA-enriched milk). **(Review) (Journal Article)** |
| (31) | (32) | (33) | (34) | (35) |
| Review of current knowledge on factors that affect consumer acceptance and ability to understand health claims (e.g., calcium-fortified) for milk compared to other products. **(Review) (Book Chapter)** | A review of milk approved as functional food across Asian legislations, including "phytosterol-enriched milk powder for heart health" by the Indonesian National Agency for Drug & Food Control, and a sterol-containing milk drink is approved to bear a label claim ‘An animal study shows that consumption of this product may help lower blood total cholesterol’ by the Taiwanese Health Food Control Act". (Review) (Book Chapter) | Review of qualified (e.g., "milk is very important for our health") and unqualified well-being messaging for milk according to Autocontrol Spanish advertising regulatory body. (Review) (Journal Article) | A proposed category of functional dairy beverages. A comprehensive list of commercial functional milk beverages (probiotic, omega-3 enriched, with added bioactive components, and added minerals and vitamins) with information on milk type, components used, brand, manufacturer, manufacturing process, and claims made. (Review) (Journal Article) | A review of the actions toward promoting organic food, including the nutritional benefits claims of organic compared to non-organic milk, made by proponents of organic food (i.e., two non-government organizations, the Soil Assn. in the United Kingdom and the Organic Center in the United States). **(Review) (Journal Article)** |
| (36) | (37) | (38) | (39) | (40) |
| A list of nutrition claims (i.e., low fat milk) and conditions applying to them according to European Union regulations. **(Review) (Journal Article)** | Approved and disapproved examples of well-being messages for milk in accordance to the Swiss Federal Court, e.g., milk helps "to prevent bone fragility at older age, socalled osteoporosis" (not allowed) and "With milk you become tall and strong. And it remains" (Allowed). (Review) (Journal Article) | Review of other studies comparing pricing of flavored milk carrying GMO-free and Organic labels with regular or conventional (unlabeled) milk. (Review) (Journal Article) | A list of commercial fermented and non-fermented probiotic milk drinks and their claimed benefits in the United Arab Emirates. (Case Report) (Journal Article) | A list and technologies related to drinkable milk as a smart food with supplemented elements (e.g., calcium-enriched, selenium-rich milk powder, A2 beta-caesin variant milk, and selenium-enriched milk). Information on advantages of other ruminants' milks compared to bovine milk to be used as smart foods. **(Review) (Journal Article)** |
| (41) | (42) | (43) | (44) | (45) |
| A list of commercial milk as a functional food with supplemented element and health claims (e.g., Becel pro.activ (with plant sterols), blood cholestrol level, and LGG Plus milk drink, Bowel comfort). A discussion of regulations in Sweden. **(Review) (Journal Article)** | Proposed nutrient profiling system according to European regulations and the eligibility of milk (i.e., semi-skimmed milk) as a carrier of well-being messaging compared to other products. (Conceptual Piece) (Journal Article) | Review of consumer understanding and the importance of nutrition and health claims particularly calcium content (natural versus added) for milk with different fat content versus yoghurt. (Review) (Journal Article) | Using health claims for milk considering the new Australia New Zealand Health Claim Standard (e.g., the A2 milk health claim "prevent childhood diabetes and heart disease") was deemed illegal). Prevalence of general, specific, or implied health claims for milk on the Australian market. (Review) (Journal Article) | A comprehensive food labeling compliance guide to understand the requirements of the Food and Drug Administration (FDA) relating to various aspects of milk and other food product characteristics, product name, content, allergens, labeling, use of claims, etc. (e.g., fat content, and freshness). **(Review) (Book)** |
| (46) | (47) | (48) | (49) | (50) |
| Uses milk as an example to explain various claim types (i.e., health claims, structure/function claim, and nutrient content claim) according to FDA regulations. **(Review) (Journal Article)** | A brief introduction to Japanese Functional Food regulations, including "milk powder for pregnant or lactating women" as one of the five food categories for "Special Health Use" under the Japanese Nutrition Improvement Law. (Review) (Book Chapter) | A list of globally manufactured commercial functional foods (including milk), the manufacturer, country, and composition. (Review) (Journal Article) | European regulations regarding well-being messaging of milk. (Review) (Journal Article) | A list of globally manufactured commercial fermented and non-fermented functional milk drinks, their bioactive components, and claimed benefits and in specific conjunction to Australasian market opportunities, challenges, and regulations. **(Review) (Journal Article)** |
| (51) | (52) | (53) | (54) | (55) |
| Review of milk as a functional food, functional milk-derived ingredients, and related potential health benefits and claims. **(Review) (Journal Article)** | A list of commercial functional foods, including milk drinks, with approved health use, their body functions, bioactive components, clinical trials, and qualified health claims, in conjunction to Japanese market opportunities, challenges, and regulations and other regulatory systems. (Review) (Journal Article) | Review of literature regarding differences in level of contaminants, quality, content of desirable ingredients and suitability for cheese making and sensory properties between organic and conventionally produced milk. (Review) (Book Chapter) | Lists food claims (e.g., fortified with phytosterol, n-3 fatty acids, n-3 and n-6 fatty acides from vegetable oil, and probiotics) approved or disapproved for milk in different Latin American countries. (Review) (Journal Article) | Review of the organization and implementation of nutrition policies and examine intervention strategies for dietary change in three Scandinavian countries, with specific mention of keyhole symbol labeling regulations for milk in Sweden. **(Review) (Journal Article)** |
| (56) | (57) | (58) | (59) | (60) |
| Swedish regulations for FOP key-hole symbol labeling of milk. **(Review) (Journal Article)** | A political analysis of producer (i.e., the United States organic milk industries)-consumer (i.e., consumers of organic milk) discourse on promoting organic milk. (Conceptual Piece) (Journal Article) | Labeling requirements of milk according to European Union regulations. (Review) (Book Chapter) | Regulations for milk labelling in the United States. (Review) (Book) | FDA regulations regarding well-being messaging for milk in comparison to other regulations (e.g., FOSHU in Japan, and European regulations). Provides a few examples of milk with functional benefits. **(Review) (Journal Article)** |
| (61) | (62) | (63) | (64) | (65) |
| Well-being messaging for milk from European and US federal food regulations point of view. **(Review) (Journal Article)** | Accuracy and understandability of milk fat content labels and issues regarding the method to motivate the consumer to use information and act on it. (Review) (Journal Article) | Misleading use of dairy products, particularly whole milk, from the freedom to make health claims in accordance to the U.S. regulatory systems to sell high fat products by claiming that it is high in calcium and can reduce the risk of osteoporosis prior to Nutrition Labeling and Education Act (NLEA) in 1990. The U.S. food label and health claims regulatory systems pre- and post-Nutrition Labeling and Education Act (NLEA) in 1990. (Opinion Paper) (Journal Article) | Two particular cases of unqualified claims regarding milk powder (i.e., "[t]he use of Meadow Fresh [a powdered, dairy-based drink] instead of milk will reduce the incidence of cardiovascular disease due to reduced levels of xanthine oxidase"), and milkshake (i.e., "[o]ur sodium is down across the menu" and "[w]holesome milk, natural sweeteners, a fluid ounce of flavoring, and stabilizers for consistency. And that's all") have been discussed. (Review) (Journal Article) | Examples of the health claims for milk which would be legal or illegal according to the United Kingdom food regulations. **(Review) (Journal Article)** |
| (66) | (67) | (68) | (69) |  |
| Examples of the health claims for milk which would be legal or illegal according to the United Kingdom food regulations. **(Review) (Journal Article)** | Regulation of health claims for milk (e.g., general claim (e.g., "everybody needs milk"), calorie reduction claim (e.g., "skim milk for that skim trim look")) according to various legislation authorities in the United States. (Review) (Journal Article) | Opinions of the Deputy Director, Bureau of Enforcement, Food and Drug Administration, United States Department of Health, Education and Welfare regarding proper versus what is proclaimed for milk and dairy products, including examples of claims based on vitamins and mineral content, and weight control, among others and in accordance to FDA regulations. (Opinion Paper) (Journal Article) | Suggestions on how to promote milk. (Opinion Paper) (Journal Article) |  |

**References**

1. Chalamaiah MU, S. K.: Hong, H.: Wu, J. P. Regulatory requirements of bioactive peptides (protein hydrolysates) from food proteins. *J Funct Foods* (2019) 58:123-9.

2. von Philipsborn PS, Jan M: Burns, Jacob: Busert, Laura K: Pfadenhauer, Lisa M: Polus, Stephanie: Holzapfel, Christina: Hauner, Hans: Rehfuess, Eva. Environmental interventions to reduce the consumption of sugar-sweetened beverages and their effects on health. *Cochrane Db Syst Rev* (2019) (6).

3. Brown HMR, M. E.: de Vlieger, N. M.: Collins, C. E.: Bucher, T. Influence of the nutrition and health information presented on food labels on portion size consumed: a systematic review. *Nutr Rev* (2018) 76(9):655-77.

4. Dolgopolova IT, R. Consumers' willingness to pay for health benefits in food products: A meta-analysis. *Appl Econ Perspect P* (2018) 40(2):333-52.

5. Kuczora S. Authorised EU health claim for meal replacements. *Foods, Nutrients and Food Ingredients with Authorised EU Health Claims*. 3. Dudley Metropolitan Borough Council, Dudley, United Kingdom: Elsevier Inc. (2018). p. 179-200.

6. Steinhauser JH, U. Consumer and product-specific characteristics influencing the effect of nutrition, health and risk reduction claims on preferences and purchase behavior - A systematic review. *Appetite* (2018) 127:303-23.

7. Collier RJX, Y: Bauman, D. E. Regulation of factors affecting milk yield. In: Watson RRC, R J: Preedy, V R, editor. *Nutrients in dairy and their implications for health and disease*. University of Arizona, Mel and Enid Zuckerman College of Public Health and School of Medicine, Arizona Health Sciences Center, Tucson, AZ, United States: Elsevier Inc. (2017). p. 3-17.

8. Coutrelis NR-R, Lise. Focus on the French experiment to indicate the origin of milk and meat on some food products labeling. *Eur Food Feed Law Rev* (2017) 12(1):57-8.

9. Kilara A. Regulatory aspects of yogurt. In: Shah NP, editor. *Yogurt in health and disease prevention*. Nutri Food Business Consultants, Chapel Hill, NC, United States: Elsevier (2017). p. 107-32.

10. Marinangeli CPFH, James D. Potential impact of the digestible indispensable amino acid score as a measure of protein quality on dietary regulations and health. *Nutr Rev* (2017) 75(8):658-67.

11. Zafar MZH, N. A.: bin Halim, F. The pivotal role of user-friendly food label and personality traits on intention to consume packaged food products. *J Food Prod Mark* (2017) 23(7):835-56.

12. Brody T. Food and dietary supplement package labeling-Guidance from FDA's Warning Letters and Title 21 of the Code of Federal Regulations. *Compr Rev Food Sci F* (2016) 15(1):92-129.

13. Little MC. Yes, the FDA can make you say that: Why the FDA's proposed nutrition facts label changes will withstand first amendment challenges from food industry members. *Indiana Health Law Rev* (2016) 13(1):233-71.

14. Nielsen KE. Health beneficial consumer products-status and trends. In: Osborne S, Morley, W, editor. *Developing food products for consumers with specific dietary needs*. Nutrition Business Strategies Ltd, United Kingdom: Elsevier Inc. (2016). p. 15-42.

15. Aryee ANAB, J I. Current and emerging trends in the formulation and manufacture of nutraceuticals and functional food products. In: Boye JI, editor. *Nutraceutical and functional food processing technology*. 1st ed. Agriculture and Agri-Food Canada, Saint-Hyacinthe, Canada: John Wiley & Sons, Ltd. (2015). p. 1-63.

16. Kurajdova KT-P, Janka. Literature review on factors influencing milk purchase behaviour. *Int Rev Manag Mark* (2015) 5:9-25.

17. Rimpeekool WS, S. A.: Banwell, C.: Kirk, M.: Yiengprugsawan, V.: Sleigh, A. Food and nutrition labelling in Thailand: a long march from subsistence producers to international traders. *Food Policy* (2015) 56:59-66.

18. Corbo MRB, A.: Petruzzi, L.: Casanova, F. P.: Sinigaglia, M. Functional beverages: The emerging side of functional foods commercial trends, research, and health implications. *Compr Rev Food Sci F* (2014) 13(6):1192-206.

19. McPherson MJ. What's in a name: the Vermont Genetically Engineered Food Labeling Act. *J Law Biosci* (2014) 1(3):359-68.

20. Natterer A. Monsterbacke: A judgment of only historical significance? *Eur Food Feed Law Rev* (2014) 9(4):247-9.

21. Ohama HI, H: Moriyama, H. Health foods and foods with health claims in Japan. *Nutraceutical and functional food regulations in the United States and around the World*. Biohealth Research Ltd., Tokyo, Japan: Elsevier Inc. (2014). p. 265-99.

22. Zawistowski J. Regulation of functional foods in selected Asian countries in the Pacific Rim. In: Bagchi D, editor. *Nutraceutical and functional food regulations in the United States and around the World: Second edition*. Elsevier Inc. (2014). p. 419-63.

23. Liu RDP, Z.: Verbeke, W. Consumers' attitudes and behaviour towards safe food in China: A review. *Food Control* (2013) 33(1):93-104.

24. Midtvedt T. The probiotic concept: Will it survive? *Microb Ecol Health Dis* (2013) 24:12-.

25. van't Riet J. Sales effects of product health information at points of purchase: A systematic review. *Public Health Nutr* (2013) 16(3):418-29.

26. Alibabić VM, Ibrahim: Rudić, Dušan: Bajramović, Melisa: Jokić, Stela: Šertović, Edina: Ruţnić, Alma. Labeling of food products on the B&aH market and consumer behavior towards nutrition and health information of the product. *Procedia Soc Behav Sci* (2012) 46:973-9.

27. Hastak MM, M. B. Deception by implication: A typology of truthful but misleading advertising and labeling claims. *J Public Policy Mark* (2011) 30(2):157-67.

28. Heggum C. Labeling of dairy products. In: Fuquay JW, editor. *Encyclopedia of dairy sciences*. 2nd ed. Danish Agricultural and Food Council, Aarhus N, Denmark: Elsevier Inc. (2011). p. 1-8.

29. Kaur SD, M. Functional foods: An overview. *Food Sci Biotechnol* (2011) 20(4):861-75.

30. Korhonen HJ. Functional foods : Concept to product. In: Saarela M, editor. *Functional foods: Concept to product*. 2nd Edition ed. Cambridge: Woodhead Publishing (2011). p. 471-511.

31. Lähteenmäki L. Consumers and health claims for functional foods. In: Saarela M, editor. *Functional foods: Concept to product*. Woodhead Publishing Limited (2011). p. 109-26.

32. Zawistowski J. Legislation of functional foods in Asia. In: Saarela M, editor. *Functional foods: Concept to product*. Woodhead Publishing Limited (2011). p. 73-108.

33. Melchor SRT, Liesbeth. Article 10(3) of Regulation (EC) 1924/2006: The road to salvation? *Eur Food Feed Law Rev* (2010) 5(1):22-7.

34. Özer BHK, Huseyin Avni. Functional milks and dairy beverages. *Int J Dairy Technol* (2010) 63:1-15.

35. Rosen JD. A review of the nutrition claims made by proponents of organic food. *Compr Rev Food Sci F* (2010) 9(3):270-7.

36. Verhagen HV, E.: Francl, S.: Heinonen, M.: van Loveren, H. Status of nutrition and health claims in Europe. *Arch Biochem Biophys* (2010) 501(1):6-15.

37. Zbinden KK. F(G)ood advertising practice in Switzerland. *Eur Food Feed Law Rev* (2010) 5(6):341-6.

38. Drichoutis ACL, P.: Nayga, R. M. On consumers' valuation of nutrition information. *B Econ Res* (2009) 61(3):223-47.

39. Senok AC. Probiotics in the Arabian gulf region. *Food Nutr Res* (2009) 53(1):1842-.

40. Bermingham ENR, N. C.: Anderson, R. C.: Barnett, M. P. G.: Knowles, S. O.: McNabb, W. C. Smart Foods from the pastoral sector: Implications for meat and milk producers. *Aust J Exp Agr* (2008) 48(6-7):726-34.

41. Asp N-GN-GB, Susanne. Health claims in the labelling and marketing of food products: The Swedish food sector's Code of Practice in a European perspective. *Scand J Food Nutr* (2007) 51(3):107-26.

42. Labouze EG, C.: Azais-Braesco, V. "TheFoodProfiler": A Nutrient Profiling system to restrict the use of nutrition and health claims to foods with desirable nutrient profiles. *Sci Aliment* (2007) 27(6):413-22.

43. Leathwood PDR, D. P.: Strater, P.: Todd, P. M.: van Trijp, H. C. Consumer understanding of nutrition and health claims: sources of evidence. *Br J Nutr* (2007) 98(3):474-84.

44. Lederman J. The challenge for the dairy industry arising from the new Health Claims Standard: an impetus for innovation. *Aust J Dairy Technol* (2007) 62(2):95-9.

45. Summers JLC, Elizabeth J. *Food labeling compliance review*. Ames, Iowa, USA: Blackwell Publishing Professional (2007) 2007-08. 1-318 p.

46. Agarwal SH, S.: Morar, S. Nutritional claims for functional foods and supplements. *Toxicology* (2006) 221(1):44-9.

47. Zawistowski J. Food regulations: Health claims for foods fortified with carbohydrates or other nutraceuticals. In: Biliaderis CG, Izydorczyk, M S, editor. *Functional food carbohydrates*. Functional Foods and Nutraceuticals, Forbes Medi-Tech, Inc., Vancouver, BC, Canada: CRC Press (2006). p. 527-60.

48. Arvanitoyannis ISVH-K, M. Functional foods: a survey of health claims, pros and cons, and current legislation. *Crit Rev Food Sci Nutr* (2005) 45(5):385-404.

49. Cheftel JC. Food and nutrition labelling in the European Union. *Food Chem* (2005) 93(3):531-50.

50. Sharma R. Market trends and opportunities for functional dairy beverages. *Aust J Dairy Technol* (2005) 60(2):195-8.

51. Playne MJB, L. E.: Smithers, G. W. Functional dairy foods and ingredients. *Aust J Dairy Technol* (2003) 58(3):242-64.

52. Shimizu T. Health claims on functional foods: the Japanese regulations and an international comparison. *Nutr Res Rev* (2003) 16(2):241-52.

53. Kouba M. The product quality and health implications of organic products. In: Kyriazakis I, Zervas, G, editor. *Organic meat and milk from ruminants*. (2002). p. 57-64.

54. Lajolo FM. Functional foods: Latin American perspectives. *Br J Nutr* (2002) 88 Suppl 2:S145-50.

55. Roos GL, M.: Anderson, A. Dietary interventions in Finland, Norway and Sweden: nutrition policies and strategies. *J Hum Nutr Diet* (2002) 15(2):99-110.

56. Bruce A. Strategies to prevent the metabolic syndrome at the population level: role of authorities and non-governmental bodies. *Br J Nutr* (2000) 83:S181-S6.

57. DuPuis EM. Not in my body: rBGH and the rise of organic milk. *Agr Hum Val* (2000) 17:285-95.

58. Love DA. Labelling requirements: European Union. In: Blanchfield JR, editor. *Food labelling*. CRC Press (2000). p. 31-47.

59. Altman TA. *FDA and USDA Nutrition Labeling Guide : Decision diagrams, check*: CRC Press (1998) 1998.

60. Berner LAOD, J. A. Functional foods and health claims legislation: Applications to dairy foods. *Int Dairy J* (1998) 8(5-6):355-62.

61. Kernon JS, L. Current issues in European and US federal food regulations. *Trends Food Sci Technol* (1993) 4(7):203-9.

62. O’Donnell JA. Future of milk fat modification by production or processing: Integration of nutrition, food science, and animal science. *J Dairy Sci* (1993) 76(6):1797-801.

63. Silverglade BA. A comment on "Public policy issues in health claims for foods". *J Public Policy Mark* (1991) 10(1):54-62.

64. Hobbs COM, D. B. Health claims: What food marketers should know about current Ftc, Nad, State, and Lanham Act precedents. *Food Drug Law J* (1988) 43(2):223-48.

65. Kirk T. Food marketing: Nutrition and health claims. *Nutr Food Sci* (1988) 88(4):8-9.

66. Kirk TRA, U. Legislation and codes of practice: Nutrition information in food marketing. *Br Food J* (1988) 90(6):268-72.

67. Hutt PB. Government: Regulation of health claims in food labeling and advertising. *Food Drug Law J* (1986) 41(1):3-73.

68. Milstead KL. Milk and other dairy products : What is proclaimed what is proper to proclaim. *Food Drug Cosmet Law J* (1963) 18:584-93.

69. Anderson ZE. Use of nutrition information in promoting the use of milk and milk products. *J Dairy Sci* (1954) 37(4):467-71.
